# Can Any Procedure Be Hypnosis? Exploring the Effect of Framing on Hypnotic Depth and Electrophysiological Correlates of Hypnosis in a Balanced Placebo Design

**DOI:** 10.1111/psyp.70183

**Published:** 2025-11-07

**Authors:** Zoltan Kekecs, Endre Csikos, Nguyen Dang Quy Minh, Yeganeh Farahzadi, Peter Simor, Balazs Nyiri, Pietro Rizzo, Jay A. Olson, Gary Elkins

**Affiliations:** ^1^ Institute of Psychology ELTE, Eötvös Lorand University Budapest Hungary; ^2^ Psychology MA Programme ELTE, Eötvös Lorand University Budapest Hungary; ^3^ Doctoral School of Psychology, Institute of Psychology ELTE, Eötvös Loránd University Budapest Hungary; ^4^ Institute of Behavioural Sciences Semmelweis University Budapest Hungary; ^5^ Psychology—Master of Science Programme Lund University Lund Sweden; ^6^ Department of Psychology University of Toronto Mississauga Mississauga Ontario Canada; ^7^ Baylor University Waco Texas USA

**Keywords:** hypnosis, labeling, psychophysiology, socio‐cognitive theory

## Abstract

Expectancy theory of hypnosis posits that any procedure can serve as a hypnotic induction provided it is labeled as “hypnosis”. The present study explored this hypothesis by contrasting the effects of two conventional and two unconventional (placebo) hypnotic inductions on hypnotic experiences and electrophysiological correlates. In a 2 × 2 balanced placebo design, all participants were exposed to four conditions: conventional induction labeled as “hypnosis”, conventional induction labeled as “control”, unconventional induction labeled as “hypnosis”, and unconventional induction labeled as “control”. EEG was recorded from 61 channels. We computed EEG features that were identified in previous studies as correlates of hypnosis or hypnotizability. Consistent with the predictions of expectancy theory, we found that labeling of the procedure was most influential in determining subjective hypnosis depth, and one of the unconventional (placebo) inductions, “white noise hypnosis”, evoked comparable hypnosis depth to the conventional hypnotic inductions. However, contrary to its predictions, “embedded hypnosis”, another unconventional induction, evoked smaller hypnosis depth reports than the other three inductions. Both relaxation and embedded induction procedures showed decreased gamma power in the midline occipital area. Most EEG features we explored were comparable between conventional and unconventional induction conditions, but labeling also seemed to have no effect on EEG changes, which is contrary to the prediction of the expectancy theory. Possible exceptions were a negative effect of conventional induction on functional connectivity between the O1‐Pz channels in the theta band, and a decrease in anterior and posterior alpha power in trials labeled “hypnosis”. However, these effects were inconclusive. Overall, our results provide only partial support for the expectancy theory of hypnosis. The most promising EEG correlate of hypnosis based on our results is decreased occipital gamma power. However, our findings should be considered exploratory. Confirmatory research is required to strengthen our confidence in these effects.

**Trial Registration:**

The research plan of this study was registered on the Open Science Framework (https://osf.io/kugdf) before any data collection. The data collection of the first 9 participants was not in accordance with the research plan due to a programming error. The data from these trials was not used in the confirmatory analysis. Consequently, we made changes to the sampling plan and the analysis plan. The altered version of the research plan had been registered at the Open Science Framework (https://osf.io/wvhda) before any new data was collected

## Abstract 2

Framing is thought to be a key ingredient in inducing hypnosis. This study provides a novel exploration of how procedures framed as “hypnosis” differ from those framed as non‐hypnotic relaxation in electrophysiological activity. Our balanced placebo design separates the effect of labeling from the effect of procedural differences among different unconventional and conventional induction techniques. Our results also verify previous findings about electrophysiological correlates of hypnosis.

## 1 Introduction

Hypnosis has been studied in both clinical practice and cognitive neuroscience, offering unique insights into the nature of consciousness and cognitive control. In clinical settings, hypnotherapy has demonstrated efficacy in managing a wide range of conditions, including chronic pain (Langlois et al. 2022), anxiety and mood disorders (Milling et al. 2019; Valentine et al. 2019), irritable bowel syndrome (Schaefert et al. 2014), and postoperative side effects (Kekecs et al. 2014). Beyond its therapeutic applications, hypnosis serves as a powerful experimental tool in cognitive neuroscience, allowing researchers to manipulate subjective experiences and cognitive processes in controlled laboratory settings (Halligan and Oakley 2013; Terhune et al. 2017). Moreover, research regarding hypnotizability has provided valuable insights into individual differences in cognitive flexibility and top‐down control mechanisms (Dienes et al. 2022).

Hypnotic inductions are communications used to help induce hypnotic phenomena (Edmonston 1991). More specifically, most inductions are implemented with the intention to create an attentional state in which the participant is highly attentive and absorbed in the words of the hypnotist, while being inattentive to external stimuli (often with the ultimate goal to increase responsiveness to therapeutic suggestions) (Barber 1984; Terhune and Cardeña 2016). However, although hypnotic induction is most often employed with the above goals, empirical findings do not support its effectiveness in achieving them. Though there is some evidence suggesting that participants have an increased suggestibility after a hypnotic induction, this effect seems to be small and is possibly explained by differences in expectancy and motivation (Braffman and Kirsch 1999; Kirsch and Braffman 2001). Thus, the value and specificity of classical hypnotic inductions are questioned by advocates of the socio‐cognitive theories of hypnosis, arguing that their primary effect lies in conveying response expectancies and contextual response sets (Lynn et al. 2008; Spanos 1986). It follows from this argument that basically any procedure that is convincingly labeled as “hypnosis” could be as effective as conventional inductions.

It is important to ascertain whether hypnotic inductions have any specific effective components, because if they do, we can isolate and potentially enhance them to improve effectiveness. On the other hand, if it turns out that a wide range of procedures can serve as effective inductions regardless of procedural features, we could shift resources from perfecting the details of inductions to optimizing expectancy and contextual factors and examine the mechanisms through which expectations exert their cognitive and phenomenological effects.

One line of research addressing this question explores the effect of the label “hypnosis” on hypnotic responses. For example, Glass and Barber (1961) found that a higher level of responsiveness to suggestion was obtained when the same experimental situation was labeled to participants as “hypnosis” rather than as a “control” procedure. Evidence also suggests that presenting no formal induction at all, but labeling the procedure as “hypnosis” anyway, is as good as having the formal induction. For example, Lynn et al. (2002) found no difference in responses to suggestions between two versions of the Carleton University Responsiveness to Suggestion Scale (CURSS), one presented with hypnotic induction, the other without induction, but both presented as hypnosis. These findings support other similar reports by Barber and Calverley (1964, 1965), and by Krystek and Kumar (2016) who showed that simply labeling an experimental situation as “hypnosis” without actually including a conventional hypnotic induction increased responsiveness to suggestions. These studies suggest that a hypnotic induction can lose its effect if it is relabeled as non‐hypnotic, and a situation with no conventional induction can gain the effects of formal induction simply by relabeling it as “hypnosis”, lending credit to the claim that the only necessary component of a hypnotic induction is the (credible) label of “hypnosis”. Some research does suggest that a hypnotic induction might indeed be necessary for responding to certain types of suggestions, such as more difficult, cognitive suggestions, or to elicit psychophysiological changes (Connors et al. 2012; Derbyshire et al. 2004, 2009). However, in these studies, the hypnotic induction is contrasted to a non‐hypnotic control condition. Thus, they are unable to test the claim that the only necessary component of a hypnotic induction involves setting hypnosis‐related response expectancies and context.

Studies using “placebo” or “sham” hypnotic inductions are better for evaluating the claim that anything identified as an induction can serve as one. These studies include some procedures labeled as “hypnosis” that lack the commonly identified components of hypnotic inductions, such as suggestions for focused attention, absorption, or reduced critical thinking (Brown et al. 2001; Terhune and Cardeña 2016), while matching common nonspecific components of hypnotic inductions. For example, Baker and Kirsch (1993) found that analgesic suggestions were equally effective when presented after standard hypnotic induction or after taking a placebo pill described as “a hypnotic drug that increases suggestibility”. Another study reported contradictory results (Council et al. 1983). In this study, the researchers used fake theta biofeedback as a placebo hypnotic induction, and contrasted the effects with different conventional (trance) and unconventional (skills) inductions. The placebo induction yielded slightly lower behavioral suggestibility scores on the Stanford Hypnotic Susceptibility Scale, Form C (SHSS:C) than the other induction groups, and slightly lower hypnosis depth than the trance induction group. However, there was no significant difference in the SHSS:C subjective responses ratings nor on the Creative Imagination Scale (CIS). A recent study corroborates some of these previous findings (Kekecs et al. 2024): analgesic suggestions were equally effective when received following a conventional relaxational hypnotic induction or a “white noise” placebo hypnotic induction. However, the subjective hypnosis depth was rated slightly lower in the placebo condition. Other studies also hint at a possible interaction between the effect of the label “hypnosis” and the specific components of the technique, showing that the “hypnosis” label works better when attached to some procedures (e.g., relaxation) rather than others (Barber 1969; Barber and Calverley 1964, 1965; Gandhi and Oakley 2005). However, there is little research on such moderating effects. To be able to disentangle the effect of the label “hypnosis” from the effect of the specific components of the induction, and to be able to assess potential interactions, in our present study, we use a balanced placebo design. In these designs, what participants are told they are receiving is fully crossed with what they actually receive. For example, participants either receive a medication or a placebo while being told that it is either a medication or a placebo, resulting in four conditions. Here, conventional and unconventional (placebo) inductions are either labeled as highly effective hypnotic induction techniques or non‐hypnotic control techniques.

An important limitation of prior research on the necessary components of hypnosis induction is that almost all studies used measures that can be influenced by the participant either consciously or unconsciously, for example, due to demand characteristics or expectations. This also limits the impact of previous research on the theoretical debate. If a study finds supporting evidence for or against the prediction that any procedure can serve as hypnosis induction, skeptics can dismiss the findings on the grounds that the results can be explained by bias from participant expectations and insufficient blinding. Thus, a crucial test of the prediction of the socio‐cognitive theory also needs to involve objective measures. If there are differences in the effectiveness of different hypnotic induction procedures, we can expect to find differences in the psychophysiological correlates of hypnosis as well.

Many studies have investigated the psychophysiological correlates of hypnosis (Landry et al. 2017). Some evidence implicates the importance of alpha power in frontal and posterior regions (De Pascalis 1993; Graffin et al. 1995), although, the localization and direction of change are not clear (Hiltunen et al. 2021; Jensen et al. 2015; Landry et al. 2024; Sabourin et al. 1990). Decreased gamma power was also found in previous studies to be correlated with deep hypnosis reports (Hiltunen et al. 2021; Hinterberger et al. 2011). However, another study found that faster cortical activity, including gamma power, was associated with greater hypnotic effects (Cardeña et al. 2013). Some studies also found that frontal and posterior theta power increased during hypnotic induction both in low and high hypnotizable participants (Sabourin et al. 1990; Williams and Gruzelier 2001), but see Hiltunen et al. (2021) who found no such effect. Furthermore, some evidence suggests that fronto‐parietal phase synchrony in the alpha2 band (10.5–12 Hz) is decreased during hypnosis among highly hypnotizable subjects (Terhune et al. 2011). Another research group found a decrease in functional connectivity between occipital and parietal hubs in the beta1 (13–19.9 Hz) and theta bands to be associated with hypnotic rest (Jamieson and Burgess 2014). While these reports give some hints about potential electrophysiological correlates of hypnosis, the lack of consistency across findings limits confidence in them. Thus, it is not yet possible to pinpoint a single electrophysiological correlate of hypnosis. Instead, we have to first conduct an exploratory study to understand which electrophysiological measure is the best candidate for a future crucial test of the theory.

In summary, the primary goal of our present study was to evaluate the claim derived from the expectancy theory of hypnosis that any procedure may serve as a hypnotic induction as long as the right hypnosis‐related expectancies and context are provided. The expectancy theory predicts that after controlling for contextual effects (such as the labeling of a procedure as “hypnosis”) and expectancy, there will be no subjective or physiological differences between various induction procedures in their hypnotic effects.

In this paper, we will focus on two main research questions: What are the differential effects of procedural characteristics of inductions and labeling, in both (1) hypnotic depth experiences reported by participants, and (2) electrophysiological correlates of hypnosis? Answering these questions is essential to be able to design a confirmatory test of the expectancy theory of hypnosis. We used a balanced placebo design to be able to disentangle the differential role of the label “hypnosis” and the induction type. Due to uncertainties in our understanding of the effects and convincingness of the unconventional induction techniques we intended to use, as well as about the psychophysiological correlates of hypnosis, this study was designed as exploratory.

## 2 Methods

### 2.1 Sample

Participants were recruited among university students via flyers and via email announcements. We also advertised the study on Facebook groups involving people interested in psychology. Participants received supermarket vouchers worth ~20 € if they completed both research sessions. Some participants also received course credit for their participation.

We chose our sample size target (52 eligible participants) for this exploratory study for pragmatic reasons based on what we thought would be possible in a realistic timeframe for this research. Nevertheless, we conducted a simulation‐based power analysis before data collection to understand the operational characteristics of our study relative to our confirmatory hypothesis. Power to support the null hypothesis was 77%, while power to support the alternative hypothesis was 85%. The sample size as well as the power analysis results were preregistered. Details are presented in the Supplement.

### 2.2 Procedures

The study consisted of two research sessions. In the first session, participants underwent a pre‐experimental closed‐eye rest period, followed by 4 experimental trials, and ending with a post‐trial rest period. EEG was recorded throughout this first session. In the second research session, participants underwent a hypnotizability measurement.

In the first session, after the participants gave informed consent, they underwent a standardized rapport conversation about hypnosis with an experimenter. Subsequently, the EEG cap was mounted (see “EEG measurements” below). Once the EEG cap was set up, the experimenter left the room, and the participant began the session using the OpenSesame stimulus‐presentation software (Version 3.3.8; Mathôt et al. 2011). In the OpenSesame application, participants first filled out a pre‐experimental questionnaire that gathered demographic information, motivation to get hypnotized, general attitudes toward hypnosis, and past experiences and knowledge about hypnosis. Participants then underwent a pre‐hypnosis baseline measurement, where they closed their eyes and rested in a chair for 5 min in silence, while EEG activity was recorded. After this baseline rest period, they read a brochure about hypnosis, after which they participated in four experimental trials.

#### 2.2.1 Experimental Trials

In each of the four experimental trials, participants experienced a different hypnotic induction procedure. Two of these procedures were conventional hypnotic induction methods (relaxation induction and confusion induction), while the other two were unconventional (“placebo”) inductions (white noise induction and embedded induction). The description of these induction procedures can be found in the Supplement. All recordings were matched in length and sound level. The audio recordings and scripts are available on OSF.

##### 2.2.1.1 Labeling

Among these four induction procedures, two were labeled as “hypnotic inductions” which had been found effective for inducing deep hypnosis, while the other two were labeled as “control” procedures that may help with relaxation but could not induce hypnosis. Labeling (“hypnosis” or “control”) was determined randomly with the restriction that one of the conventional hypnosis procedures was always labeled as “control” and one of the unconventional procedures was always labeled as a “hypnotic induction”. This produced a 2 × 2 balanced placebo design (2 induction types × 2 labels), where every participant was exposed to four conditions: a conventional hypnotic induction labeled as hypnosis, a conventional induction labeled as control, an unconventional (placebo) induction labeled as hypnosis, and an unconventional (placebo) induction labeled as control. The order in which the conditions were presented and their labeling as either hypnosis or control was randomized in the software, so that experimenters were also effectively blind to this information.

During each trial, before the induction procedure began, participants read the description of the upcoming induction procedure and then rated their expectation of how deeply hypnotized they expected to become during this technique, on a scale ranging from 0 (Not Hypnotized at All) to 10 (Extremely Hypnotized).

Participants then listened to a 6‐min recording of the induction procedure. Upon completion of the induction, participants heard the prompt, “for the next several minutes, just continue to experience the state you are in right now,” and they sat quietly with closed eyes for 5 min. This was followed by a hypnosis de‐induction. After each induction procedure, participants rated how deeply hypnotized they felt they were during the 5‐min hypnotic rest period on the same 0 to 10 scale as before. Participants were asked to describe their experiences during the 5‐min hypnotic rest period using their own words.

All the study materials are shared on OSF: https://osf.io/prscg/files/osfstorage.

#### 2.2.2 Post‐Trial Period

After the experimental trials, participants underwent another resting state EEG measurement similar to the pre‐hypnosis baseline measurement. We do not use data from this measurement in this paper.

At the end of the experiment, participants completed a post‐experimental questionnaire, which included a question about their preferred effective hypnosis technique. Here they were asked to choose one of the two hypnosis techniques (the two techniques labeled as “hypnosis”) in response to the following question: “If you have to undergo a dental procedure that is likely to be painful, and if hypnotic induction would be the only type of analgesia available for you, which of the two hypnotic induction techniques would you choose?”

The questionnaire also contained a de‐briefing that one of the hypnosis techniques used was not an evidence‐based hypnotic induction and that one of the control techniques was, in fact, an evidence‐based hypnotic induction. They were asked whether they had suspected this and to identify which technique labeled as “control” was actually an evidence‐based induction, and which technique labeled as “hypnosis” was truly so.

#### 2.2.3 EEG Measurement

Brain electrophysiological activity was obtained throughout the first research sessions using the BrainAmp Standard amplifiers and Standard 128Ch BrainCap Sleep from Brain Products with 128 built‐in Ag/AgCl passive ring electrodes including EEG, EOG, and EMG electrodes. We used 61 of the 128 channels. Details are provided in the Supplement.

#### 2.2.4 Second Research Session

In this session, we assessed hypnotizability using the Harvard Group Scale of Hypnotic Susceptibility (HGSHS) (Shor and Orne 1962), which is the group version of the Stanford Hypnotic Susceptibility Scale, Form A (Weitzenhoffer and Hilgard 1959). This self‐scored measure rates hypnotizability on a scale of 0 to 12. Additionally, participants were interviewed about their perception of hypnosis and their experiences during the experimental trials.

### 2.3 Data Analysis

The EEG data was processed with Python 3.9. All other data analysis was done in R (Version 4.4.1 (R Core Team 2024)).

#### 2.3.1 EEG Preprocessing

All preprocessing steps were done using the Python‐based MNE package v1.0.3 (Gramfort et al. 2013). Due to considerable developments in the field since our preregistration (2021), we have used a slightly different preprocessing plan to better fit currently accepted best practices.

We extracted each experimental trial's data from raw continuous data and organized them into BIDS format with MNE‐BIDS (v0.10; Pernet et al. 2019). Subsequently, we inspected the data visually to detect electrodes with poor signals and reconstructed them using spline interpolation based on neighboring electrodes. At this step, we also inspected the data visually to assess the types of artifacts present. In total, 0.36% of all channels across all participants were marked as poor.

The following preprocessing steps were automated for reproducible results and the dispersion vector (Bigdely‐Shamlo et al. 2020) was used at the end of each of these preprocessing steps to check the quality of the data. In this automated pipeline, the data was first high‐pass filtered at 1 Hz and stop‐band at 42 Hz using a zero‐phase finite impulse response filter with a Hamming window. Then the eye movement artifacts were removed separately for each experimental trial using the implementation of the FastICA algorithm and CORRMAP method (Campos Viola et al. 2009) in the MNE package. With CORRMAP, three components were selected which represented typical blink and eye movement artifacts and used them as a template for selecting and excluding similar components for other participants' data. Afterward, the continuous data was segmented into windows of 1000 ms. At this point, remaining artifacts (e.g., drifts, head movements, or transient jumps) were rejected automatically using Autoreject (v0.3.1; Jas et al. 2017) so that those epochs that were contaminated with these transient artifacts were excluded (7.8% of all the epochs in total). Subsequently, the signal was re‐referenced to the average of the electrodes. Ultimately, the segmented data was transformed into a continuous format.

#### 2.3.2 EEG Features of Interest

We analyzed EEG features identified in previous research as potential correlates of hypnosis. Specifically, we refer to the following variables as EEG features of interest in the following analyses:

- The change in EEG power in the theta (4 to < 8 Hz), alpha (8 to < 13 Hz), and gamma (> 30 to 42 Hz) bands in the midline frontal (FPz, Fz) and midline occipital (POz, Oz, Iz) regions. We will refer to these regions in the tables and figures as FzArea and OzArea, respectively. These frequency bands and regions were selected based on (Cardeña et al. 2013; De Pascalis 1993; Graffin et al. 1995; Hiltunen et al. 2021; Hinterberger et al. 2011; Jensen et al. 2015; Sabourin et al. 1990; Williams and Gruzelier 2001).
- The change in functional connectivity between the Oz and PO4 channels in the beta1 (13 to < 20 Hz) band. This EEG feature and localization were selected based on (Jamieson and Burgess 2014).
- The change in functional connectivity between the midline parietal (CPz, Pz) and midline frontal (FPz, Fz) regions in the alpha2 (10.5 to 12 Hz) band. We will refer to these regions in the tables and figures as PzArea and FzArea, respectively. This EEG feature and localization was selected based on (Terhune et al. 2011).
- The change in functional connectivity between the O1‐Pz channels in the theta (4 to < 8 Hz) band. This EEG feature and localization was selected based on (Jamieson and Burgess 2014).

“Change” values are computed as the value measured during the baseline rest period subtracted from the value measured during the hypnosis rest period. Thus, positive values indicate a higher value during hypnosis. The values were *Z*‐transformed.

We calculated EEG power using Welch's method with an 8‐s Hamming window (0.125 Hz frequency resolution). We then averaged each subject's spectra across time and space within the specified frequency bands and brain areas. Frequency band boundaries are in accordance with OHBM standards (Pernet et al. 2020), with alpha2 and beta1 boundaries based on previous hypnosis studies showing significant changes after induction (Jamieson and Burgess 2014; Terhune et al. 2011).

To account for the 1/*f* power law scaling and enable comparisons of power values across experimental conditions and electrodes, we normalized absolute power values to the pre‐hypnosis baseline using *Z*‐score transformation. The *Z*‐score was calculated as:

$$Z_{\mathrm{tf}}=\frac{P_{\mathrm{tf}}\;-\;\underline{P_{\text{baseline},f}}}{\mathrm{SD}\left(P_{\text{baseline},f}\right)}$$

where $P_{\mathrm{tf}}$ represents the power at a specific time‐frequency point, $\underline{P_{\text{baseline},f}}$ is the mean baseline power for that frequency, and $\mathrm{SD}\left(P_{\text{baseline},f}\right)$ is the standard deviation of the baseline power for that frequency (Cohen 2014). This baseline, consisting of a 5‐min recording, was of sufficient duration to reduce the impact of noise and outliers, ensuring reliable estimates of the mean and standard deviation.

We calculated functional connectivity using weighted Phase Lag Index (wPLI), a measure of phase synchronization that is reliable in minimizing spurious connectivity due to the limited spatial resolution of EEG (Vinck et al. 2011). To further reduce volume conduction effects, we applied a spherical spline surface Laplacian filter before computing connectivities. For midline frontal–parietal alpha2 connectivity, we averaged ‘Fz’, ‘Fpz’, and ‘Pz’, ‘CPz’ channels to get FzArea and PzArea values, then computed wPLI between these averaged signals. All wPLI calculations were performed using the MNE‐Connectivity (v0.5.0) package.

### 2.4 Statistical Analysis

#### 2.4.1 Manipulation Check

For our preregistered manipulation check, we conducted a Bayesian linear regression test to determine if the unconventional inductions used in our study evoked expectancy comparable to conventional inductions. In this analysis, we only considered trials in which the procedure was labeled “hypnosis”. This analysis was conducted according to our preregistered plan, contrasting the likelihood of observing our data under two models: Model 0 (M0) assumes that the unconventional hypnosis induction evokes comparable expected hypnosis depth to the conventional hypnosis technique, and Model 1 (M1) assumes that the unconventional induction evokes lower expectancy than the conventional one. Details of the analysis pipeline are described in the Supplement.

We considered that a more direct test of our hypothesis is to compare each unconventional procedure to the conventional procedures directly. Thus, we also ran a modified version of the above analysis, where we restricted the data to only contain one type of conventional induction compared to one type of unconventional induction. We used a similar linear mixed effect model here as well, with expectancy as the predicted outcome variable and trial type (conventional vs. unconventional) as a fixed effect predictor with participant ID as a random effect factor. We tested each conventional–unconventional induction pair separately this way, with the inference criteria being the same as for the preregistered hypothesis test.

#### 2.4.2 Main Analyses

We were interested in the effect of procedural deviations from conventional induction procedures and the effect of labeling on hypnosis, measured on subjective reports and EEG features of interest. To assess these effects, we built separate Bayesian mixed linear regression models for each dependent variable (hypnosis depth and each EEG feature of interest). All models had the following fixed effect predictors: induction type (unconventional vs. conventional), label (control vs. hypnosis), the interaction term of induction type × label, expectancy, gender (female vs. male), trial order. All models contained a single random effect term: the random intercept of participant ID. Furthermore, we ran another model where hypnotizability was also added as a fixed effect predictor to the above‐mentioned models. (We did not use this as our main model because eight participants did not return for the hypnotizability measurement session, so this analysis would decrease our power and precision on the other effects.)

In all Bayesian analyses mentioned above, we used the same priors across the board: a Cauchy distribution with a scale parameter of 1 (we also report the results of robustness tests to evaluate the influence of prior choice). The mode and 95% credible interval of the posterior distribution were computed using a thinning interval of 1, one chain, and 10,000 iterations within the chain. We also computed the Bayes factor (BF01) for each fixed effect parameter contrasting two models: M0 assuming that the predictor has no effect in the model, while M1 assuming a non‐zero effect of the predictor. Throughout this manuscript, Bayes factor (01) ≥ 3 and ≤ 1/3 were used as thresholds for declaring moderate‐level evidence for M0 (null model) and M1 (alternative model), respectively.

A number of secondary analyses were also conducted, details of which can be found in the Supplement.

#### 2.4.3 Sensitivity Analysis

To determine if potentially unsuccessful deception had any effect on our conclusions, we conducted a sensitivity analysis. For this, we re‐ran our main analyses on the subsample of trials where the participants were deceived by the deception. We compared the conclusions of these analyses to the results of our main analyses.

#### 2.4.4 Handling Missing Data

No data imputation was implemented to supplement missing data. Participants with missing data were not excluded from analysis as long as all data for the particular analysis was present and all trials were completed by the participant.

#### 2.4.5 Handling Outliers

During the exploration of the data of the EEG features of interest, we observed some extreme cases. Thus, we implemented automated outlier exclusion, where values of the raw EEG features of interest that were more than 3 SDs higher or lower than the mean were removed. This happened on the data from baseline rest and hypnotic rest periods on the trial level separately, before *Z* transformation and before we computed change scores. Out of the 1794 data points, we excluded a total of 34 values this way, which is less than 2% of the data.

## 3 Results

### 3.1 Participant Flow and Characteristics

As specified in our preregistration, a programming error was discovered after data from nine participants were collected, so we restarted data collection, excluding those nine participants. After we restarted data collection, we collected data from 52 more participants (our registered target sample size). One of the participants was excluded due to a software issue, and five were excluded because they indicated that they had attended a university lecture on hypnosis (and were thus not hypnosis‐naive), leaving a total of 46 participants in the analyzed sample.

Thirty‐four (74%) of the participants were female, and the rest were male. Most participants (72%) were in the age range of 18 to 24, 20% were between 25 and 34, 1 participant was between 35 and 44, and the remaining 3 were in the age range of 45 to 54. On average, participants reported that they had little knowledge about hypnosis (mean = 3.41, SD = 2.38, range = 0 to 9). Eight participants did not attend the subsequent hypnotizability measurement session. Average hypnotizability was 5.61 (SD = 2.38, range = 1 to 10). The attitude of participants was generally favorable toward hypnosis (mean = 2.70, SD = 1.67, range = −1 to 5); only one participant reported a negative score (−1) on the attitude scale; everyone else was either neutral (0) or positive. Most participants reported that they believed that hypnoanalgesia was effective (mean = 6.39, SD = 1.87, range = 3 to 10).

### 3.2 The Effect of Different Induction Procedures on Expectancy (Manipulation Check)

The unconventional induction procedures need to be convincing so that we are able to match hypnotic response expectancies evoked by conventional inductions. Thus, we have preregistered a hypothesis as a manipulation check: At least one of the unconventional (placebo) hypnotic induction techniques will produce comparable expectancies to at least one of the conventional hypnotic induction techniques when they are both labeled as effective hypnotic inductions. Comparable is defined as at most 1‐point difference in the averages between the expectancy scores in the unconventional and the conventional hypnotic induction trial.

Expected hypnosis depth was profoundly influenced by whether the procedure was labeled as non‐hypnotic control or hypnosis (see Table 1). Collapsed across induction procedures, on average, the hypnosis description resulted in 3.41 (SD = 2.82, BF01 < 0.001, 95% CI = 2.82, 3.96) greater expectancy rating. On the other hand, when collapsed across labels, average expectancy evoked by the different procedures lies within 1 point (means and SDs: relaxation: 4.39 [3.08], confusion: 4.98 [2.72], embedded: 4.35 [3.11], white noise: 5.33 [2.64]).

**TABLE 1:** Expectancy statistics by procedure and label.

Procedure type	Mean (SD)	# Trials
Labeled as control		
Relaxation	2.56 (2.44)	27
Confusion	3.26 (2.64)	19
Embedded	2.79 (2.73)	29
White noise	4.06 (2.68)	17
Labeled as hypnosis		
Relaxation	7.00 (1.7)	19
Confusion	6.19 (2.08)	27
Embedded	7.00 (1.54)	17
White noise	6.07 (2.36)	29

We ran a formal test of whether unconventional inductions used in this study are comparable in their evoked expectancy to conventional inductions. The inference of this Bayesian test turned out to be variable, because of the randomness involved in the statistical procedure and the Bayes factor being very close to 3 (our inference threshold for accepting the hypothesis). Thus, we ran the analysis 1000 times. The mode of the Bayes factors calculated this way was 2.93 with 12% of the Bayes factors lying above 3. This indicates that our results are inconclusive, but leaning toward supporting the hypothesis that the unconventional inductions evoked comparable expectancies to the conventional inductions.

We also ran a test comparing each unconventional procedure separately to each conventional procedure, which is a better match to our original hypothesis (this test was not preregistered) These analyses indicate that the expectancy evoked by the embedded induction is comparable to that of relaxation induction (BF = 4.69), and that white noise induction evokes comparable expectancy to confusion induction (BF = 6.12), while the other two comparisons are inconclusive.

Overall, the manipulation was successful, since the description of both unconventional hypnotic induction techniques produced comparable expectancy to at least one of the conventional hypnotic induction techniques, and mean expectancy of all four procedure types was within 1 point of each other.

### 3.3 How Do Induction Type and Labeling Affect Subjective Hypnosis Depth?

As shown in Table 2, label (hypnosis or control) was the main influencing factor of subjective hypnosis depth ratings. Very strong Bayesian evidence supported that labeling has an effect on hypnosis depth (*b* = 1.73 (0.84, 2.63), BF(01) < 0.01), supporting the prediction of the expectancy theory of hypnosis (see Table 3 for detailed regression results).

**TABLE 2:** Hypnosis depth statistics by procedure and label.

Procedure type	Mean (SD)	# Trials
Labeled as control		
Relaxation	2.78 (2.99)	27
Confusion	3.89 (2.85)	19
Embedded	1.34 (1.67)	29
Whitenoise	3.41 (3.00)	17
Labeled as hypnosis		
Relaxation	6.00 (2.16)	19
Confusion	5.63 (3.13)	27
Embedded	3.59 (3.61)	17
Whitenoise	5.41 (3.05)	29

**TABLE 3:** Regression coefficients for predicting hypnosis depth.

	*b* (95% CI)	BF (01)
Intercept	2.45 (0.07, 4.84)	NA
Induction type—conventional	1.08 (0.4, 1.75)	0.09
Label—hypnosis	1.73 (0.84, 2.63)	0.01
Expectancy	0.26 (0.08, 0.43)	0.09
Gender—male	0.19 (−1.06, 1.46)	4.97
Trial number	−0.18 (−0.49, 0.12)	4.25
Induction type × label	−0.13 (−0.79, 0.52)	6.25

*Note:* *b* indicates posterior mode of the regression coefficient based on 1 chain and 10,000 iterations; 95% CI represents 95% credible intervals.

**FIGURE 1:**
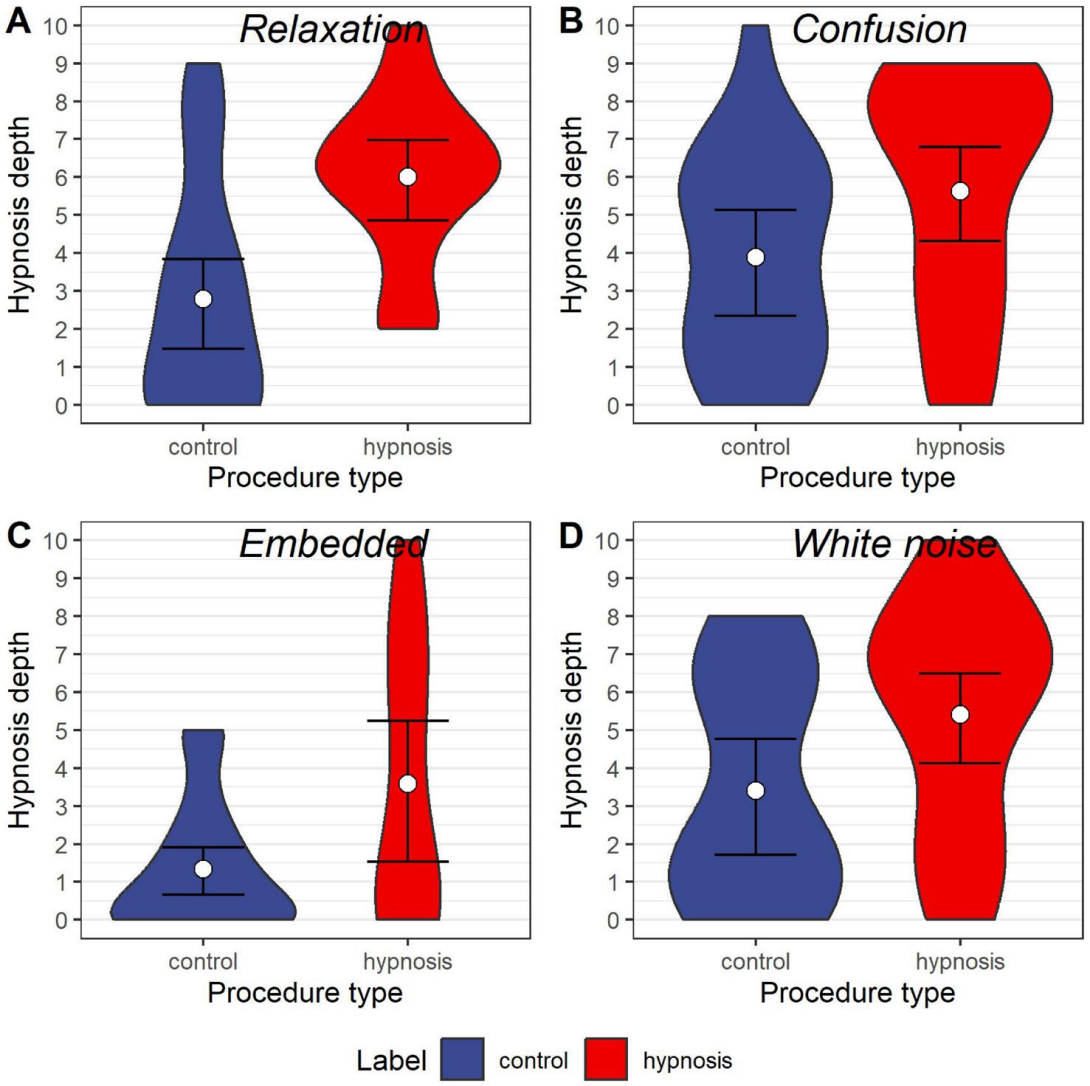
Distribution of hypnosis depth ratings by procedure type. The figure displays the distribution of hypnosis depth ratings by procedure type. Violin plots filled blue represent procedures labeled as “control”, while those colored red represent procedures labeled “hypnosis”. Whiskers represent 95% credible intervals. Panels A and B represent data of conventional induction procedures (relaxation and confusion, respectively), while panels C and D represent data of unconventional induction procedures (embedded and white noise).

At the same time, contrary to the expectancy theory of hypnosis, strong Bayesian evidence supported that the type of induction (conventional vs. unconventional) affected hypnosis depth (*b* = 1.08 (0.40, 1.75), BF(01) = 0.09) even after controlling for the effect of labeling and expectancy in the model. As shown in Figure 1, the main driver of this effect seems to be that participants exposed to the embedded hypnotic induction condition reported on average more than two points lower hypnosis depth compared to the other procedures (irrespective of labeling). There was no interaction between labeling and induction type (*b* = −0.13 (−0.79, 0.52), BF(01) = 6.25).

### 3.4 How Do Induction Type and Labeling Affect EEG Features of Interest?

None of the models provided substantial Bayesian evidence for the effect of labeling on any of the EEG features of interest (all BFs > 0.33). All but two models provided moderate‐level evidence supporting no effect of labeling on the EEG feature we investigated (BFs > 3). The two exceptions were the models predicting change in FzArea and OzArea alpha power. We found a small negative effect of the “hypnosis” label on these EEG features, but the Bayesian evidence was inconclusive (alpha: FzArea: *b* = −0.28 (−0.52, −0.05), BF (01) = 0.44; alpha: OzArea: *b* = −0.29 (−0.57, −0.03), BF (01) = 0.82; regression coefficients for these models are shown in Table 4).

**TABLE 4:** Regression coefficients for predicting EEG features of interest.

	*b* (95% CI)	BF (01)
FzArea alpha power		
Intercept	0.22 (−0.62, 1.05)	NA
induction type—conventional	−0.13 (−0.31, 0.05)	4.91
Label—hypnosis	−0.28 (−0.53, −0.04)	0.60
Expectancy	0.04 (−0.01, 0.09)	1.69
Gender—male	0.07 (−0.44, 0.59)	3.77
Trial number	0 (−0.08, 0.08)	12.15
Induction type × label	0.07 (−0.11, 0.24)	5.95
OzArea alpha power		
Intercept	0.27 (−0.57, 1.12)	NA
Induction type—conventional	−0.08 (−0.28, 0.12)	8.82
Label—hypnosis	−0.29 (−0.56, −0.02)	0.85
Expectancy	0.03 (−0.02, 0.09)	3.76
Gender—male	−0.25 (−0.76, 0.24)	2.84
Trial number	0 (−0.09, 0.09)	10.68
Induction type × label	0.03 (−0.17, 0.23)	8.26
Connectivity—O1‐PZ—theta band		
Intercept	0.1 (−0.74, 0.96)	NA
Induction type—conventional	−0.23 (−0.42, −0.05)	0.59
Label—hypnosis	0.15 (−0.09, 0.4)	4.36
Expectancy	−0.04 (−0.1, 0.01)	1.65
Gender—male	−0.1 (−0.65, 0.43)	4.35
Trial number	0 (−0.08, 0.08)	10.34
Induction type × label	0.09 (−0.09, 0.28)	5.27

*Note:* *b* indicates the posterior mode of the regression coefficient based on 1 chain and 10,000 iterations; 95% CI represents 95% credible intervals. The table is segmented based on the dependent variable used in the model. This table only includes the results of three of the nine models. For the full table, see Table S1.

**FIGURE 2:**
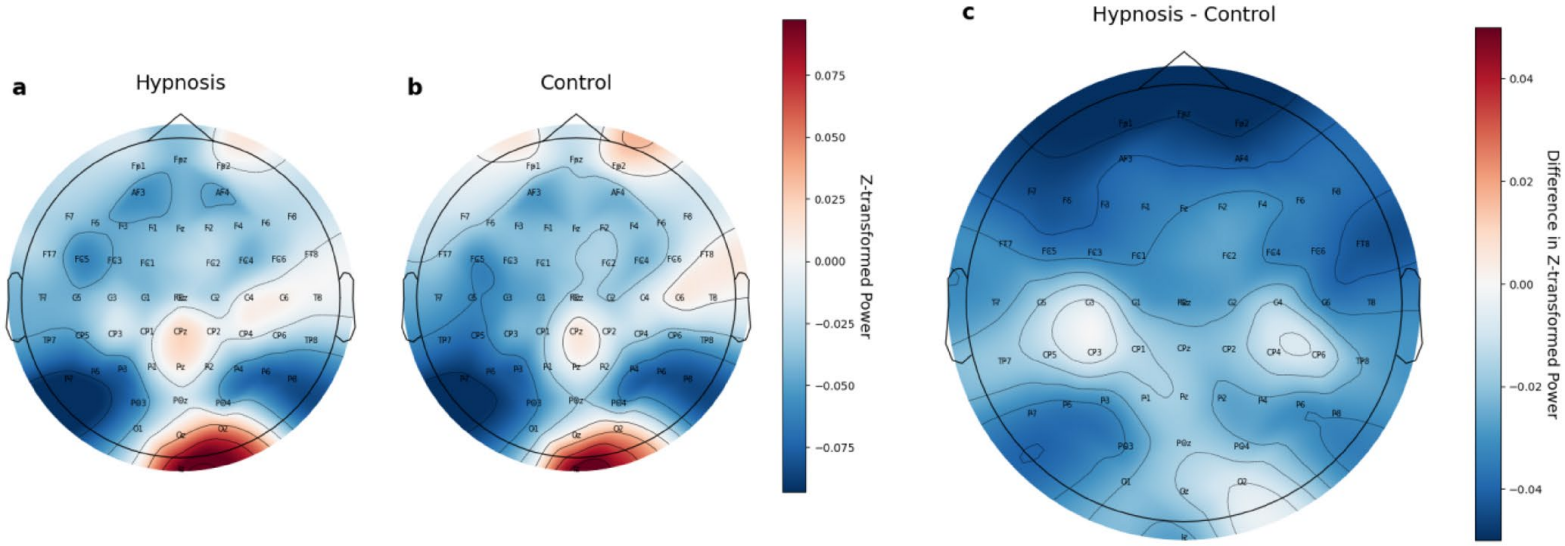
The topological graph of changes in the alpha band in trials labeled as hypnosis and control. This figure compares the topography of *z*‐transformed alpha power normalized to the pre‐hypnosis baseline (baseline1) values for both the trials labeled as hypnosis (panel a) and as control (panel b), along with their mean difference (panel c). The data are averaged across participants to highlight overall patterns.

**FIGURE 3:**
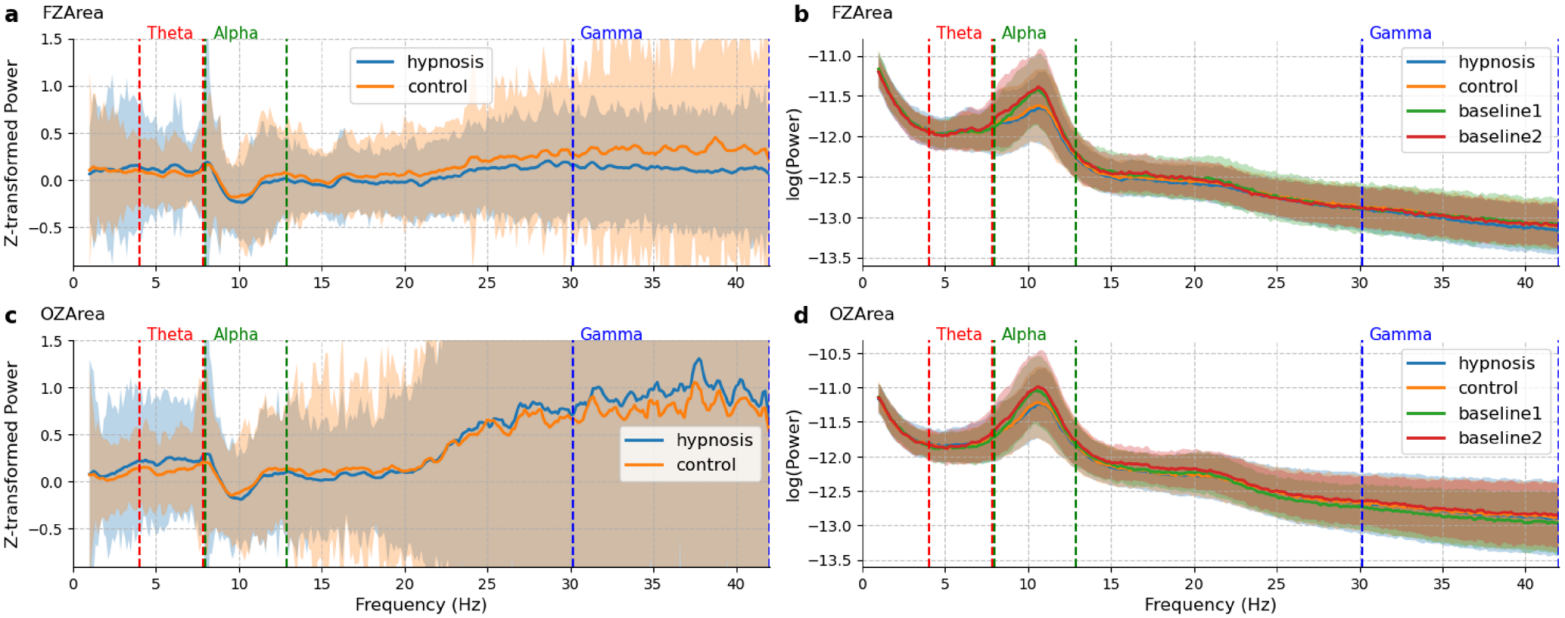
Power spectral density at pre‐and post‐hypnosis baselines and in trials labeled as hypnosis and as control. This figure compares power spectral density (PSD) data for two conditions (trials labeled as hypnosis or control) at the FZ and OZ areas. Panels a and c show *z*‐transformed PSD values normalized to the pre‐hypnosis baseline (baseline1) for both conditions, while panels b and d display logarithmic transformed power values. Each curve represents the average across participants, with standard deviation envelopes indicating variability.

A topological graph (Figure 2) and power spectral density distribution (Figure 3) are shown for the alpha band to illustrate this potential effect. Interestingly, the graphs of normalized PSD (Figure 3, panels b and d) indicate that on average alpha power at both midline frontal and midline occipital areas was lower in the experimental trials compared to the baseline rest conditions, irrespective of induction type (conventional vs. unconventional), and label (hypnosis vs. control). These differences compared to baseline do not seem to be related to the experimental conditions, and are present in conventional induction trials labeled as hypnosis just as well as unconventional inductions labeled as control (Figure S1, panels b and d). Thus, it is likely that these are the effects differences between the baseline and the experimental rest conditions, rather than being hypnosis effects. We discuss the implications of these effects for psychophysiological research in the Discussion section.

**FIGURE 4:**
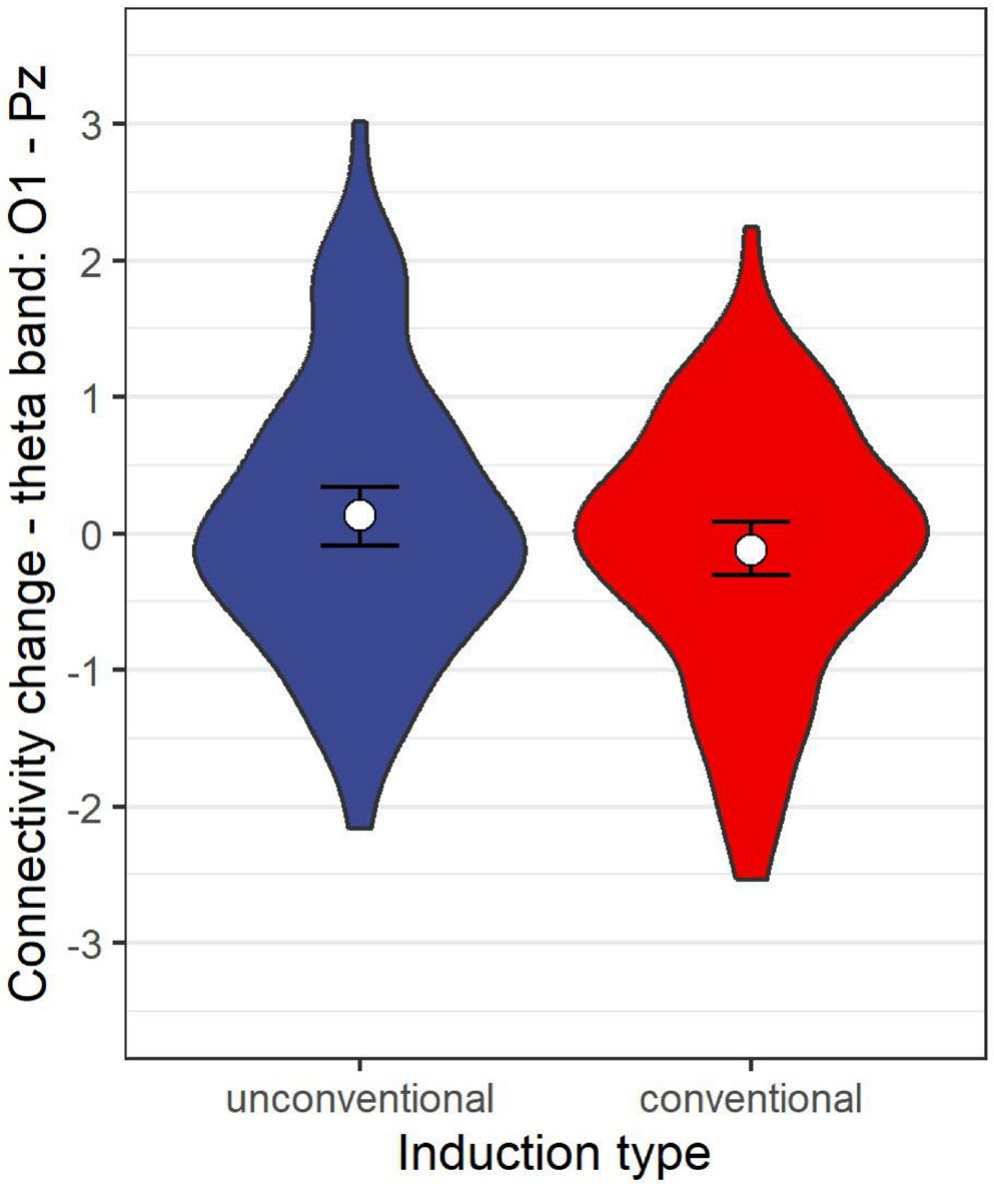
Change in functional connectivity between the O1‐Pz channels in the theta band. The figure displays the distribution of change in functional connectivity between the O1‐Pz channels in the theta band for each induction type. The violin plot filled blue represents trials with unconventional hypnosis induction procedures, while the one colored red represents trials with conventional induction procedures. The white disk shows the posterior mode, while whiskers represent 95% credible intervals.

Similarly, none of the models provided substantial Bayesian evidence supporting the effect of induction type (all BFs > 0.33). In fact, all but one model provided moderate to strong Bayesian evidence supporting that there is no effect of induction type on the EEG features of interest (BFs > 3), with one exception: we found a slight negative effect of conventional hypnosis on the functional connectivity between the O1‐Pz channels in the theta band, but the Bayesian evidence was inconclusive (*b* = −0.23 (−0.42, −0.05), BF = 0.59; details of regression coefficient are shown in Table 4). See Figure 4.

None of the effects of the other covariates or predictors entered into the models to predict the EEG features of interest were supported by substantial Bayesian evidence (all BFs > 0.33). Regression coefficients of all predictors in all models are reported in Table S1.

We also explored adding the effect of hypnotizability to the models. Hypnotizability had a positive influence on hypnosis depth but it did not show an effect on any of the EEG features of interest (all BFs > 1), and adding hypnotizability to the models did not change the other effects substantially (see Table S2).

### 3.5 Secondary Analyses

#### 3.5.1 Procedure‐Level EEG Changes

We investigated the baseline to hypnotic rest changes in the EEG features of interest for each induction procedure separately. In this analysis, we only take into account trials which were labeled as hypnosis. We found moderate to strong Bayesian evidence supporting decreased gamma power in the midline occipital area in relaxation induction trials (posterior mode: −0.28 (−0.44, −0.08), BF(01) = 0.15) and the embedded hypnosis induction trials (posterior mode: −0.27 (−0.41, −0.08), BF(01) = 0.09). In all other procedures and EEG features, Bayesian evidence was leaning toward supporting the null (all BFs > 1), and in most cases, moderate Bayesian evidence supported the null model, indicating no substantial change in the EEG features of interest. Details are reported in Table S3.

#### 3.5.2 Prediction Accuracy of EEG Features Combined for Induction Type

We also built a mixed‐effects logistic regression model to see if the EEG features of interest together are informative in predicting which induction type (conventional or unconventional) was used in the trial. We found that all EEG features of interest taken together were not effective at distinguishing between conventional and unconventional inductions, since they produced comparable prediction accuracy on the original and permuted samples. Bayesian evidence was inconclusive about the fit of this logistic regression model (BF01 = 0.94). The details of this analysis can be found in the Supplement.

#### 3.5.3 Correlation of EEG Changes With Hypnosis Depth and Hypnotizability

We analyzed the relationship between hypnosis depth and hypnotizability and the change in the EEG features of interest where the procedure was labeled as hypnosis to the subjects. In general, correlations were small and 95% credible intervals always overlapped with 0 (see Tables S4 and S5). In most cases, moderate Bayesian evidence (3 < BF01s < 10) supported the absence of correlation between the EEG features of interest in hypnosis depth or hypnotizability. We also built linear regression models to predict hypnosis depth and hypnotizability by including all EEG features of interest as predictors in trials where the procedure was labeled as hypnosis. Both the analyses using permutation testing and Bayes Factors (BF01s > 10) revealed very poor prediction performance, suggesting no substantial relationship between the studied EEG features and hypnotic depth or hypnotizability. The detailed results of these analyses are presented in the Supplement.

#### 3.5.4 Sensitivity Analysis

In order to assess the effect of unsuccessful deception on our conclusions, we conducted a sensitivity analysis by rerunning our main analyses on the subsample of trials in which participants appeared to be deceived by the deception. This consists of 100 (54%) trials (for the detailed numerical results on deception effectiveness and the sensitivity analyses, see the Supplement).

The conclusions of these analyses are largely the same as those in the full sample. Expectancy was comparable in all four induction procedures, and it was highly dependent on the label: trials labeled as control evoked much lower expectancy than trials labeled as hypnosis. The preregistered hypothesis test was significant in this subsample, BF = 5.53, supporting M0. The main regression analyses on the effect of induction type and labeling on hypnosis depth and the EEG features of interest also showed similar results to those we got on the full sample (see Table S6). There was one slight difference that is worth mentioning: we found moderate Bayesian evidence for no effect of induction type on hypnosis depth (BF01 = 6.40), whereas in the full sample, conventional hypnosis had a slight positive effect on hypnosis depth.

It is important to note that the sensitivity analysis involves much less data than the main analysis, and since most of the results are consistent with those of the main analysis, we use the main analysis as the basis of our final conclusions.

#### 3.5.5 Robustness Analysis

All of our analyses reported so far used a Cauchy distribution with a scale parameter of 1. Other commonly used choices for the scaling parameter are sqrt(2)/2 and sqrt(2) leading to narrower and wider prior distributions, respectively. We investigated how sensitive the conclusions drawn from our main analyses are to the choice of prior distribution, and found that our conclusions remain the same irrespective of these prior settings.

## 4 Discussion

The current study aimed to explore the effects of labeling and induction procedural differences on subjective hypnotic experiences and psychophysiological changes during hypnosis.

First we confirmed that our manipulation was successful in that procedures that we labeled as “hypnosis” evoked greater expected hypnotic depth. This effect of the label “hypnosis” on expectancy did not seem to depend on whether the procedure was conventional or unconventional. Thus, our manipulation worked as expected: when people were told they will get hypnosis they expected much greater hypnosis depth than when they were told they will undergo a non‐hypnotic control procedure. Our preregistered confirmatory analysis was inconclusive, but another confirmatory test that better fits our original hypothesis found comparable evoked expectancy between embedded (unconventional) and relaxation (conventional) inductions and between white noise (unconventional) and confusion (conventional) inductions. This latter result confirmed the original hypothesis, that the descriptions of the unconventional induction techniques used in our research can evoke comparable expectancies to the descriptions of conventional inductions. These results are not surprising, since we worked with hypnosis‐naive participants who did not have prior experiences to compare the described procedures to. Nevertheless, these are reassuring results showing that our expectancy manipulation worked as intended.

We found that the label “hypnosis” had a substantial influence on perceived hypnosis depth. This is consistent with Kirsch's response expectancy theory, which states that expectancies not only generate the suggestive effects of hypnosis but also determine the nature and level of those (Lynn et al. 2008). Most classical accounts of hypnosis mention the induction and goal‐directed suggestions as the main components of hypnosis interventions. The importance of the preparatory stage prior to the formal induction, which is sometimes referred to as rapport, preamble, or “introductory remarks”, is often neglected. However, many studies demonstrate that these introductory remarks can be profoundly important in shaping the subjects' beliefs and expectations about the upcoming intervention, and setting the right hypnotic context (see, e.g., Accardi et al. 2013; Barber 1969; Gandhi and Oakley 2005; Lynn et al. 2002; Scacchia and De Pascalis 2020). Our results clearly support these earlier findings, since the description (labeling) of either “hypnosis” or “control” served as an introductory remark, setting response expectancies, and influencing the level of hypnosis depth reported later. However, it is important to clarify that it is not the label itself that is responsible for generating hypnotic experiences. According to the expectancy theory, the beliefs (such as beliefs about expected effects of hypnosis) are what matter. In this study, the induction labels and descriptions of the induction techniques were used to manipulate these beliefs. This manipulation is ideal for research purposes because it is simple, reproducible, and standardized. However, this manipulation may not work all the time or the same way for everyone. For example, multiple participants reported substantial hypnosis depth even after unconventional inductions labeled control. Measuring the effect of our manipulations on beliefs in a qualitative manner might yield more insight about what happened in such cases.

We found contradicting results related to the effect of the different procedure types on perceived hypnosis depth. On the one hand, white noise induction evoked comparable hypnosis depth reports to those reported during conventional induction procedures. Thus, similarly to previous findings by Kekecs et al. (2024), white noise labeled as hypnosis was effective at generating deep hypnosis reports. On the other hand, during embedded hypnosis trials, participants reported smaller hypnosis depth compared to the other three procedures. Evidence from prior studies is similarly mixed on the differences between the effects of different hypnotic inductions. For example, Spanos et al. (1986) found no differences in the responsiveness of participants to the CURSS hypnotic induction and the SHSS:C induction, while Barnes et al. (2009) found that the CURSS induction produced more “hypnotic‐like” performance than the HGSGS:A and the GSHA.

Our results pose challenges to both socio‐cognitive and special process theories. From the perspective of special process theories, the lack of effectiveness of embedded hypnotic induction is easy to explain by proposing that it did not include the necessary features of a hypnotic induction for evoking a hypnotic state. However, from this perspective, it is hard to explain the effectiveness of white noise induction, which lacks any specific features commonly found in hypnotic inductions, but which evoked comparable hypnosis depth to the conventional inductions. One explanation could be that with white noise induction, we have stumbled on a new way of inducing hypnosis, for example, because the white noise facilitates focused attention. By contrast, the relative effectiveness of the white noise induction is perfectly natural from the socio‐cognitive perspective. However, the lack of effectiveness of the embedded induction is unexpected by the socio‐cognitive theories, which usually postulate that procedural details of inductions should not matter as long as expectancy, social context, etc. are matched between the procedures (Baker and Kirsch 1993; Council et al. 1996; Lynn et al. 2008). Thus, our exploratory results suggest that a simple form of the socio‐cognitive theories, the expectancy theory of hypnosis, may not be sufficient to explain the empirical findings. Instead, a more sophisticated version of the theory might be necessary. One possible explanation for our result could be that expectancy or other important beliefs about this induction technique changed during the induction process itself. Modern descendants of the socio‐cognitive theories and the predictive coding framework such as the Response Set Theory (Lynn et al. 2023), and the Simulation‐Adaptation Theory of Hypnosis (Zahedi et al. 2024) incorporate dynamical change in predictions. These newer models might be a better fit to the results we observed. Future studies could attempt to assess changes in beliefs, subjective experiences, and attentional focus during the induction process itself.

We also evaluated electrophysiological changes during the trials. The main conclusion we can draw from these analyses is that most of the EEG features of interest explored in this study were unaffected by labeling and they did not differ between conventional and unconventional hypnosis procedures. It is important to say that these EEG features were selected based on previous findings related to electrophysiological correlates of hypnosis. A special process theory of hypnosis would predict an effect of conventional vs. unconventional induction procedures, while the expectancy theory of hypnosis would predict an effect of labeling. Thus, our results did not confirm either of these predictions. There could be multiple explanations for this. One simple explanation could be that these theories are imprecise and should be replaced by more sophisticated theories or new frameworks entirely. However, we should not rush to such conclusions yet. First of all, this was an exploratory study which was not specifically powered to find these effects, so there is a possibility that small effects remained unnoticed by our research. Also we should not discount the possibility that most previous findings related to electrophysiological correlates of hypnosis are chance findings. After all many of these findings are contradictory and were not preregistered.

One correlate of hypnosis that we were able to confirm from the previous findings was a decrease in gamma power from baseline to post‐induction. Specifically, decreased gamma power was found in the midline occipital area in both relaxation induction and embedded induction trials when they were labeled as hypnosis. Our findings match previous reports in the literature of decreased gamma activation as a correlate of deep hypnosis (Hiltunen et al. 2021; Hinterberger et al. 2011). However, our results are in conflict with those of Cardeña et al. (2013) who reported that faster wave activity corresponds with greater self‐reported hypnosis depth, although that study involved pre‐selected high and low hypnotizable participants. Gamma activity is often associated with working memory use, attention, and sensory stimulation, especially conscious perception of stimuli (Bartoli et al. 2019; Jia and Kohn 2011). It is possible that the effect we found is a component of a brain state that is unique to hypnosis (i.e., a marker of the “hypnotic state”). However, it is also possible that the decrease in gamma power simply corresponds with a non‐hypnosis‐specific shift in attention and/or cognition that hypnosis‐naive people in the right context would label “deep hypnosis”, but more experienced hypnosis subjects would label differently. This alternative explanation could be tested by comparing experienced hypnosis subjects or hypnosis virtuosos with hypnosis‐naive participants. Gamma power is also sometimes viewed as an indicator of local neural activation in general rather than a marker of specific cognitive functions (Fries 2009; Jia and Kohn 2011; Scheeringa et al. 2011). So another explanation for the finding could be that decreased cortical arousal, which might be a result of more effective (or simply more prolonged) relaxation in the hypnosis condition than during the baseline condition. We also have to note that the finding about gamma power decrease was not very robust in the sense that we did not find a similar result in the frontal region, and this effect was only shown in two out of the four induction procedures we tested. A well‐powered preregistered confirmatory study is needed to confirm this effect.

We found that the label “hypnosis” was associated with decreased alpha power in both frontal and occipital regions. The Bayesian evidence for this effect was very weak; nevertheless, the credible interval for the effect did not include zero, so it might be worth considering alpha power as a potential candidate for future research on the psychophysiology of hypnosis. Several earlier studies revealed a change in alpha power in hypnotic rest, although the localization and direction of change were not consistent (for review, see Jensen et al. 2015). Some studies such as De Pascalis (1993) reported a decrease in alpha1 and alpha2 mainly in posterior regions, while Graffin et al. (1995) reported increased alpha power across frontal and posterior regions. A recent study found that instead of raw power, higher variability in alpha peak center frequency was correlated with hypnotic susceptibility during hypnosis (Landry et al. 2024) an effect widespread across the cortex, not localized to any specific region. Other studies did not find any association between alpha power change and hypnotic responses (Hiltunen et al. 2021).

In the main analyses in this research study, we contrast trials using unconventional and conventional induction procedures. In these analyses, we do not distinguish between the two unconventional inductions (‘white noise’ and ‘embedded inductions’), and similarly, we pool the data from the two conventional inductions (‘relaxation’ and ‘confusion’). This is an a priori choice based on theory. Special process theories assert that a range of procedures is able to evoke an altered state of consciousness that is often referred to as the hypnotic state. Accordingly, conventional induction procedures in clinical practice are often used interchangeably. To some extent the same is true for research studies, although some authors caution that suggestions within induction procedures themselves might shape post‐induction hypnotic phenomena (see e.g., Cardeña et al. 2013). The expectancy theory of hypnosis extends the range of equivalent induction techniques to any procedure with matching non‐specific features such as a hypnotic context, and hypnotic response expectancies. Special process theories imply that both relaxation and confusion induction would be able to induce a hypnotic state, just like expectancy theory implies that both of these procedures would be effective hypnosis induction methods. Special process theories would predict that ‘white noise’ and ‘embedded inductions’ would be both ineffective at inducing a hypnotic state, while the expectancy theories predict that given the right non‐specific elements, they would be both effective hypnosis inductions. Thus, both theories imply that there is similarity between these procedures within categories (conventional or unconventional), but expect different results between categories. This is why this design is useful for testing the core predictions of the expectancy theory of hypnosis. Nevertheless, it is important to note that differences between the effectiveness of induction procedures are rarely studied systematically with a few noteworthy exceptions, (e.g., Alarcón et al. 1999). Thus, we only have limited empirical results supporting the comparability of these induction procedures within each category. This is why our exploratory results on these procedures both in behavioral and electrophysiological effects are important, and could be used to guide future research using these procedures or a similar paradigm. Our results provide support for the comparability of the pre‐induction description of these procedures, because they seemed to evoke comparable expectancy. However, we also found that ‘embedded hypnosis’ produced substantially lower hypnosis depth reports compared to all three other procedures, which indicates that this procedure as used in our study is less effective in evoking hypnotic experiences not only between, but within category as well. It may be valuable for future studies using a similar paradigm to use more comparable techniques at least within categories.

## 5 Limitations

The exploratory nature of this study limits the confidence in our findings. Our study was not powered to find statistically significant effects in these topics, and thus we did not use formal statistical inference. Furthermore, the utilized deception in the study seemed not to be fully successful. Although pre‐trial reports of expected hypnosis depth indicated successful manipulation of expectancy, by the end of the research study about 65% of participants reported that they suspected some form of deception in this study. Relatedly, the lower hypnosis depth measured in embedded induction trials might have been the consequence of the failure of experimental manipulation. However, our sensitivity analysis only including participants who were successfully deceived to the end of the study showed similar conclusions to the analysis of the full sample. Also, our sample mostly involves university students or young adults, mainly from a Western, Educated, Industrialized, Rich and Democratic (WEIRD) population, limiting the generalizability of our findings (Hanel and Vione 2016; Henrich et al. 2010). Another notable limitation is that our sample was not preselected based on hypnotizability. This was necessary because we wanted to include hypnosis‐naive participants. However, this means that most of our sample consists of medium hypnotizable individuals. Since many other studies in the literature assessing psychophysiological correlates of hypnosis work with preselected samples of low and high hypnotizables, our results might not be fully comparable to those coming from these studies (e.g., Cardeña et al. 2013; Hiltunen et al. 2021).

Another limitation is that we used a single‐item measure of hypnosis depth. Although such a measure of hypnotic depth is commonly used in the literature, it is possible that people with a lack of experience with hypnosis will vary greatly in their responses due to having no comparison. In our study we supplemented this measure with a free‐text response about hypnotic experiences that could shed more light on the differences in the effects of the different inductions. However, the analysis of this qualitative data is outside the scope of this paper. Furthermore, our interpretation of the results is limited by expectancy being measured only at the beginning of each hypnosis trial. Finding the proper time to evaluate expectancy in hypnosis has been an essential consideration during studies (Tomé Pires et al. 2015). Previous studies had various timings in which expectancy was measured (Reategui 2020). Whereas the majority of researchers measured expectancy either before or right after the induction procedure, measuring expectancy multiple times seems to be a less common practice (see Reategui 2020 for review). In the context of the current study, it is possible that participants may have changed their expectancies related to the procedures during the inductions. We would see a clearer picture about the role of expectations if we measured them multiple times, for example, like Benham et al. (1998). However, it is important to note that continuous measurement of expectancy could have a detrimental effect on hypnotic experiences or could interfere with the expectancy manipulation.

We noted that alpha power at both anterior and posterior areas was lower in the experimental trials compared to the pre‐ and post‐hypnosis baseline rest conditions, irrespective of induction type and label. These differences are likely to be a result of differences between the baseline and the experimental rest conditions, rather than being hypnosis effects. Most likely, these are prolonged closed‐eyes and/or relaxation effects, since in the experimental trials participants have already been sitting with closed eyes and listening to relaxing audio recordings for a few minutes before the silent rest period begins, while in the baseline rest, participants do not have this pre‐rest relaxation. Since our research questions are mainly related to between‐condition comparisons, these differences are unlikely to have a major effect on our conclusions. However, these findings have some relevance for other studies conducted on psychophysiological correlates of hypnosis and similar psychological procedures and states, because many studies compare hypnosis rest results to pre‐ (and sometimes post‐) baseline rest conditions directly. If these rest conditions, and the preparations leading up to these rest conditions are not matched properly, they could lead to artifact findings. Others have also cautioned about artifacts in alpha power related to eye‐closure (Hiltunen et al. 2021). Our findings also suggest that at least some of the previous findings about alpha band correlates of hypnosis might be experimental artifacts in studies with inadequate control of rest conditions.

## 6 Conclusion

The goal of our research was to explore the plausibility of the claim by the expectancy theory of hypnosis that procedural details of inductions are irrelevant, and thus, any procedure can serve as a hypnotic induction as long as non‐specific socio‐cognitive features are matched between induction techniques. Our research was exploratory, aiming to provide data regarding the differential effects of procedural characteristics of inductions and labeling on subjective and electrophysiological effects of hypnosis, supporting future confirmatory research and theory formation. All in all, our findings provide partial support for the expectancy theory, since the most influential factor determining subjective responses to hypnosis was labeling a procedure “hypnosis”, while the procedure used for induction seemed to matter less in this regard. In particular, induction procedures evoked negligible subjective hypnosis depth reports when they were labeled as non‐hypnotic control conditions. Also, we found evidence that most EEG features marked as potential neural correlates of hypnosis were comparable between conventional and unconventional inductions. However, the findings did not unequivocally support the claim that anything passes as a hypnotic induction. The embedded hypnotic induction procedure evoked somewhat smaller hypnosis depth reports than the other inductions. We also did not find any robust differences in electrophysiological changes evoked by procedures labeled as hypnosis vs. control, which does not match the prediction of the expectancy theory. Both relaxation and embedded induction procedures resulted in a decrease in gamma power in midline occipital areas, making this a potential electrophysiological marker of hypnosis that should be investigated in future research. With less confidence, our findings also hint at the possible role of frontal and occipital alpha power change for distinguishing between procedures labeled as hypnosis vs. control, and of a decrease in functional connectivity between the O1‐Pz channels in the theta band marking a difference between conventional and unconventional inductions. However, the exploratory nature of our investigation limits the confidence in any individual finding. Confirmatory research is required to strengthen inference.

## Author Contributions

**Zoltan Kekecs:** conceptualization, methodology, software, investigation, data curation, validation, formal analysis, supervision, funding acquisition, visualization, project administration, resources, writing – original draft, writing – review and editing. **Endre Csikos:** investigation, methodology, project administration, writing – original draft, writing – review and editing. **Nguyen Dang Quy Minh:** project administration, writing – original draft, writing – review and editing. **Yeganeh Farahzadi:** data curation, formal analysis, resources, supervision, validation, writing – review and editing. **Peter Simor:** conceptualization, funding acquisition, resources, supervision, validation, writing – review and editing. **Balazs Nyiri:** resources, supervision, writing – review and editing. **Pietro Rizzo:** conceptualization, methodology, writing – review and editing. **Jay A. Olson:** conceptualization, methodology, writing – review and editing. **Gary Elkins:** conceptualization, methodology, supervision, writing – review and editing.

## Ethics Statement

Our study was approved by the Research Ethics Committee of the Faculty of Pedagogy and Psychology (Eötvös Loránd University, Budapest, Hungary, ref. no.: 2021/345).

## Conflicts of Interest

The authors declare no conflicts of interest.

## Supporting information


**Figure S1:** Power spectral density at pre‐and post‐hypnosis baselines and in conventional and unconventional trials in trials labeled as control.
**Figure S2:** The topological graph of changes in the theta band in conventional and unconventional trials.
**Figure S3:** Power spectral density at pre‐and post‐hypnosis baselines and in conventional and unconventional trials in trials labeled as hypnosis.
**Table S1:** Regression coefficients for predicting EEG features of interest.
**Table S2:** Regression coefficients for predicting EEG features of interest—with hypnotizability added as a predictor.
**Table S3:** Procedure‐level EEG changes.
**Table S4:** Correlation of hypnosis depth and hypnotizability with change in EEG power.
**Table S5:** Correlation of hypnosis depth and hypnotizability with change in functional connectivity.
**Table S6:** Regression coefficients for predicting hypnotic depth and EEG features of interest.

## Data Availability

The data that support the findings of this study are openly available on Open Science Framework at https://osf.io/prscg/, reference number: DOI 10.17605/OSF.IO/PRSCG. Raw EEG data are shared on OpenNeuro via https://openneuro.org/datasets/ds004572. Code Availability Statement: All materials (code, research materials, etc.) required to reproduce this study are also openly shared via https://osf.io/prscg/.
