# Supplementary material for: Can Any Procedure Be Hypnosis? Exploring the Effect of Framing on Hypnotic Depth and Electrophysiological Correlates of Hypnosis in a Balanced Placebo Design
Source: Psychophysiology. 2025 Nov 7;62(11):e70183. doi: 10.1111/psyp.70183 (PMC12595402; doi:10.1111/psyp.70183)
Supplement: Supplementary file 1 — Figure S1: Power spectral density at pre‐and post‐hypnosis baselines and in conventional and unconventional trials in trials labeled as control. Figure S2: The topological graph of changes in the theta band in conventional and unconventional trials. Figure S3: Power spectral density at pre‐and post‐hypnosis baselines and in conventional and unconventional trials in trials labeled as hypnosis. Table S1: Regression coefficients for predicting EEG features of interest. Table S2: Regression coefficients for predicting EEG features of interest—with hypnotizability added as a predictor. Table S3: Procedure‐level EEG changes. Table S4: Correlation of hypnosis depth and hypnotizability with change in EEG power. Table S5: Correlation of hypnosis depth and hypnotizability with change in functional connectivity. Table S6: Regression coefficients for predicting hypnotic depth and EEG features of interest. [file PSYP-62-e70183-s001.docx]

**Can any procedure be hypnosis? Exploring the effect of framing on hypnotic depth and electrophysiological correlates of hypnosis in a balanced placebo design.**

**Supplemental materials**

**Data and code availability statement:** The data that support the findings of this study are openly available in Open Science Framework at <https://osf.io/prscg/>, reference number: DOI 10.17605/OSF.IO/PRSCG. Raw eeg data are shared on OpenNeuro via <https://openneuro.org/datasets/ds004572>. Additionally, all materials (code, research materials, etc.) required to reproduce this study are also openly shared via <https://osf.io/prscg/>.

**Description of the hypnotic induction procedures**

***Relaxation induction***: The relaxation induction is one of the most commonly used induction procedures in the field, both in laboratory and clinical research as well as clinical practice. They involve focused attention and body scanning as well as suggestions for relaxation and calmness. The specific relaxation induction technique used in this study was based on the Elkins Hypnotizability Scale [(Elkins, 2014)](https://www.zotero.org/google-docs/?KlBN2o).

***Confusion induction***: Confusion induction methods are accepted methods for inducing hypnosis in research and clinical settings, although they are less commonly used than relaxation inductions. They involve focused attention and cognitive confusion and distraction. The specific induction script was based on [(American Board of Hypnotherapy and NLP, 2017)](https://www.zotero.org/google-docs/?hLecSd).

***White noise induction:*** In this induction procedure after a short explanation of what is going to happen, the subject listened to white noise in the frequency band 30 Hz - 18000 Hz, with decreased amplitude for larger frequencies (peak amplitude at 30 Hz: -32 dB, lowest at 18000 Hz: -57 dB). This is also known as a “pink noise”, which is perceived by most as softer than regular white noise. White noise is not an established hypnosis induction method, it is not used in either clinical practice or research as a formal hypnosis induction. The procedure was based on previous work where it was used as placebo hypnosis [(Kendrick et al., 2012)](https://www.zotero.org/google-docs/?TA2EB3).

***Embedded induction:*** In this procedure the subject listened to an excerpt from Encyclopedia Britannica on human muscles. Reading encyclopaedia excerpts is not an established hypnosis induction method. This recording was developed for this study.

**Power analysis**

In this simulation-based power analysis, we estimated the power of our protocol to correctly support Model 0 (M0) if that was true, and the power of our protocol to correctly support Model 1 (M1) if that was true. M0 assumes that the sham hypnosis technique labeled as hypnosis evokes comparable expected hypnosis depth to the true hypnosis technique labeled as hypnosis, while M1 assumes that the sham hypnosis technique labeled as hypnosis evokes different expected hypnosis depth than the true hypnosis technique labeled as hypnosis. This analysis was conducted with 1000 iterations and indicated that the power to correctly support M0 was 77% (where there was no difference simulated in expectancy between the sham and the true hypnosis procedures when they were labeled as hypnosis). The power was 85% to correctly support M1 if the difference in expectancy between the sham and the true hypnosis procedures when they were labeled as hypnosis was simulated to be at least 1.5 points. The SD was always set to 2.35 and the correlation of expectancy within the same label was set to 0.63. These parameters were based on our previous study results (see preliminary results section).

**EEG setup**

Brain electrophysiological activity was obtained using the BrainAmp Standard amplifiers and Standard 128Ch BrainCap Sleep from Brain Products with 128 built-in Ag/Agcl passive rings electrodes including EEG, EOG, and EMG electrodes. We used 61 of these channels: Fpz, Fp1, Fp2, AF3, AF4, Fz, F1, F2, F3, F4, F5, F6, F7, F8, FC1, FC2, FC3, FC4, FC5, FC6, T7, T8, FT7, FT8, Cz, C1, C2, C3, C4, C5, C6, CPz, CP1, CP2, CP3, CP4, CP5, CP6, Pz, P1, P2, P3, P4, P5, P6, P7, P8, TP7, TP8, POz, PO3, PO4, Oz, O1, O2, Iz, M1, M2, EOG1, EOG2, ECG placed according to the International 10–20 placement system. EEG was recorded by BrainVision Recorder software (Brain Product Inc, V. 1.21.0303) at a sampling rate of 1000 Hz without any online filtering applied. The recordings were exported to BrainVision data format and preprocessed before conducting spectral and connectivity analyses (see EEG preprocessing below).

**Statistical analysis**

**Hypothesis test.** We conducted a Bayesian linear regression test to determine if the placebo inductions used in our study evoked expectancy comparable to conventional inductions. In this analysis, we only considered trials in which the procedure was labeled “hypnosis”.

We started by exploring whether the different procedure types evoke comparable expectancy within each trial type. (That is, testing if there is evidence supporting the comparability of the expectancy evoked by the two sham hypnosis techniques, and the two true hypnosis techniques.) We built two Bayesian mixed effect linear regression models. In the “full model” we predicted expectancy with trial type (conventional vs. unconventional) and procedure type (embedded, white noise, relaxation, confusion) as fixed effect predictors, and including a random intercept of participant ID. The reduced model was the same with the exception that procedure type would not be included in the model. We first contrasted the two models using Bayes factor to see whether there is evidence that the two models are comparable. Since we did not find evidence that the reduced model and the full model were comparable, we ran our main hypothesis test on the full model.

In our main hypothesis test, we compute the Bayes Factor (M0 vs. M1) corresponding to the effect of trial type (conventional vs. unconventional) in the regression model.

We contrasted the likelihood of observing our data under the following two models: Model 0 (M0) assumes that the sham hypnosis technique labeled as hypnosis evokes comparable expected hypnosis depth to the true hypnosis technique labeled as hypnosis; Model 1 (M1) assumes that the sham hypnosis technique labeled as hypnosis evokes different expected hypnosis depth than the true hypnosis technique labeled as hypnosis.

We used an inference threshold of Bayes factor >= 3 in each Bayesian test: that is, a Bayes factor of 3 or above would support that unconventional induction techniques labeled as hypnosis evoke comparable expectations to true hypnosis (M0). This hypothesis test was preregistered, including the analysis code.

**Secondary analyses.** To assess the effect of the different induction procedures on the change of the EEG features of interest from baseline to post-induction rest, we computed the posterior mode and 95% credible intervals from Bayesian one-sample t-tests on the change values.

We also assessed the correlation between the change of the EEG features of interest and hypnotizability and hypnosis depth. Bayes factors, and posterior mode and 95% credible intervals of correlation from Bayesian correlation tests are reported.
 We also built a mixed effect logistic regression model to see if the EEG features of interest are informative in predicting which induction type (conventional or unconventional) was used in the trial. The model included all EEG features of interest as fixed effect predictors, and participant ID as random intercept. These models only included trials where the procedure was labeled as hypnosis. We supplemented this with permutation analysis, where we ran the same analyses on 10.000 permuted samples (the permutation involved randomly shuffling the induction type outcome variable) to allow for better inference taking into account overfitting. (The logistic regression model was conducted with a frequentist approach using the lme4 package due to the Bayesian variant being very computationally demanding. However, we did perform the logistic regression model using a Bayesian model using the brms pacage as well to compute Bayes Factor, and with a lower permutation sample where we confirmed the findings of the frequentist model.)

We used the same approach to analyze whether EEG features of interest can predict hypnotizability or hypnosis depth, building linear regression models to predict hypnosis depth and hypnotizability by including all EEG features of interest as predictors in trials where the procedure was labeled as hypnosis. We fit separate models on data from conventional and unconventional induction trials. A permutation analysis was run here as well, by shuffling the outcome variable, to allow for inference that is more resistant to overfitting. As with the above-mentioned analysis, we used frequentist linear models by default, but we also ran a Bayesian version of the analysis to compute Bayes factors and to confirm the frequentist inference.

**Results**

**Power spectral density**

Figure S1. Power spectral density at pre-and post-hypnosis baselines and in conventional and unconventional trials in trials labeled as control
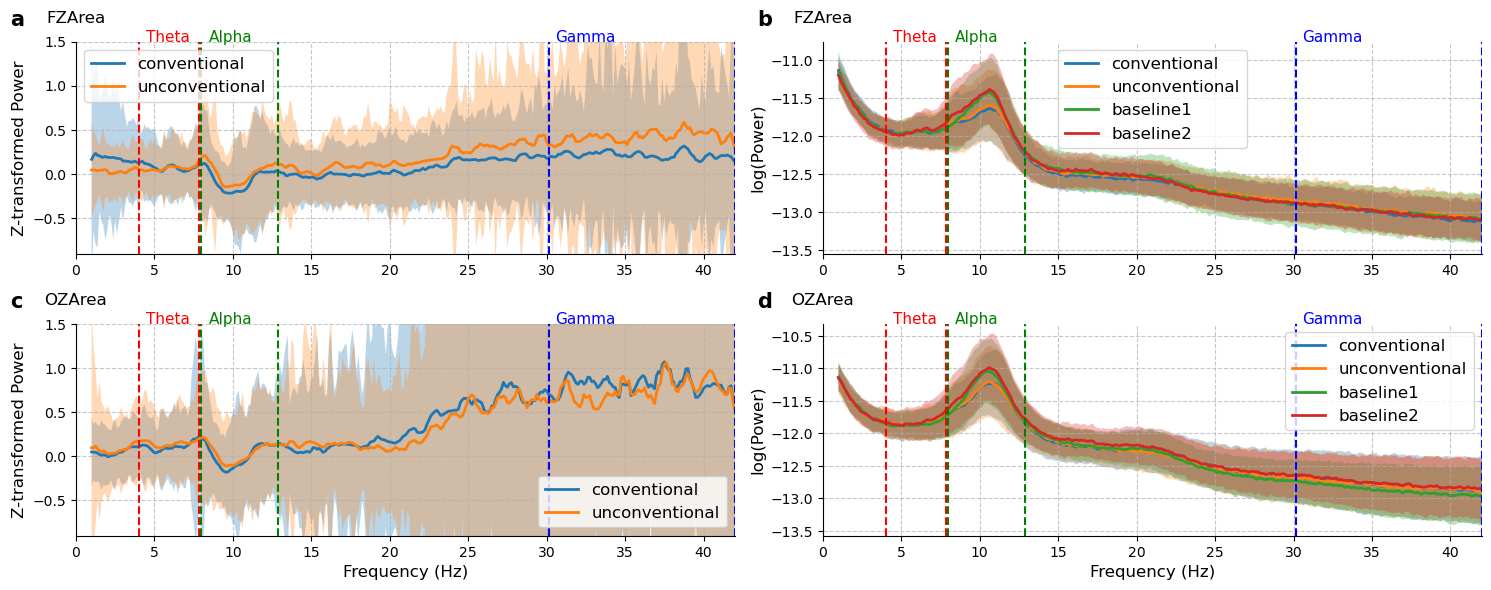


Note: This figure presents power spectral density (PSD) data during trials labeled as control, comparing conventional and unconventional conditions at the FZ and OZ electrode areas. Panels **a** and **c** display z-transformed PSD values, while panels **b** and **d** show logarithmic transformed power values.

Figure S2. The topological graph of changes in the theta band in conventional and unconventional trials


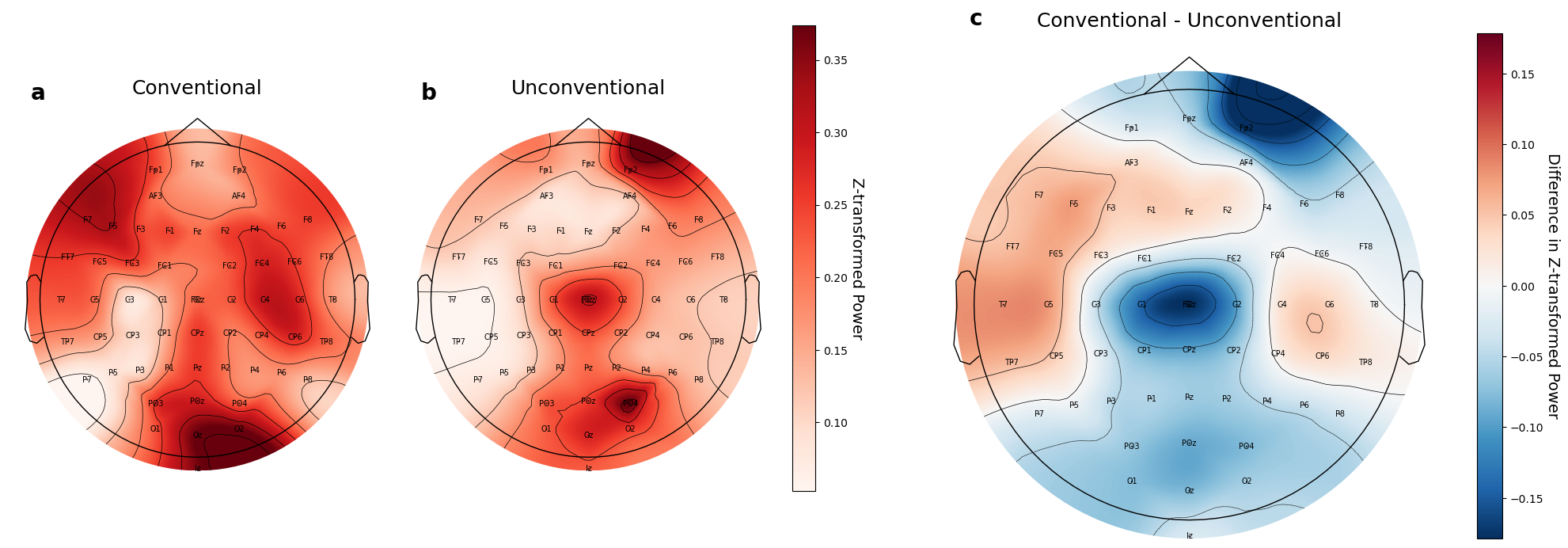


Note: This figure presents the topographical map of z-transformed theta power normalized to the pre-hypnosis baseline (baseline1) values for both the conventional (panel **a**), unconventional (panel **b**) trials, and their difference (panel **c**). The data are averaged across participants.

Figure S3. Power spectral density at pre-and post-hypnosis baselines and in conventional and unconventional trials in trials labeled as hypnosis


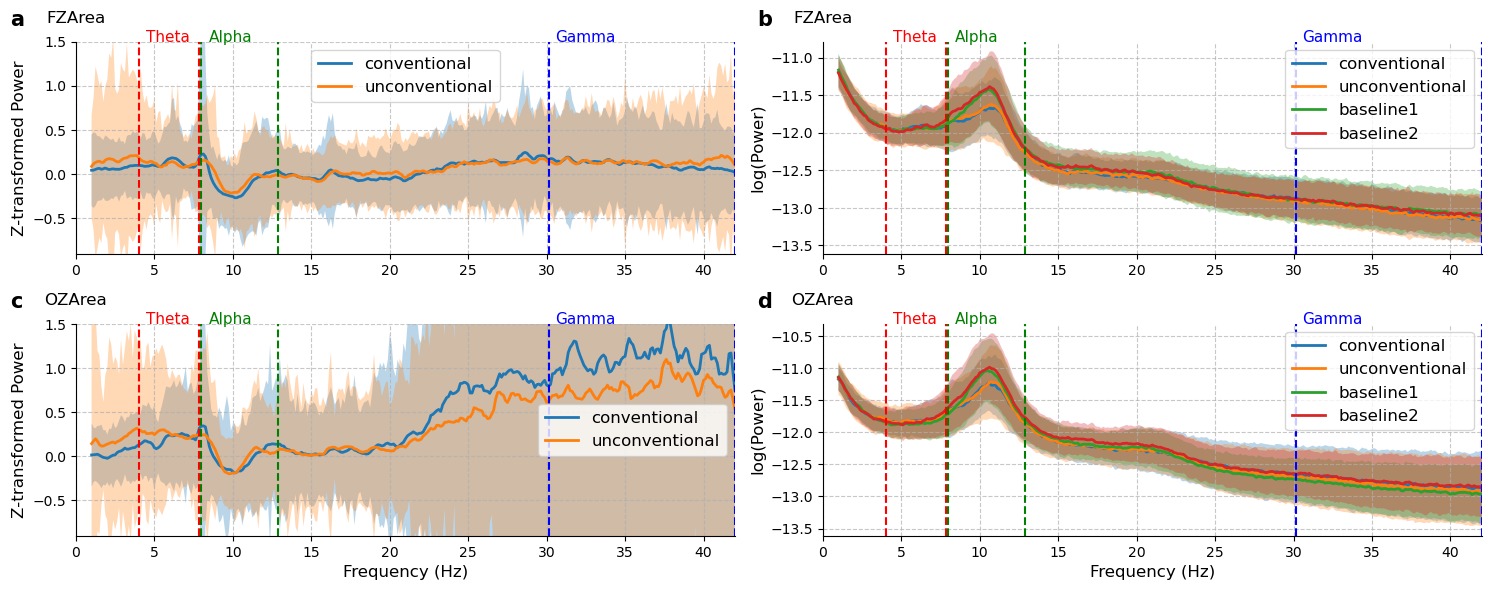


Note: This figure presents the power spectral density (PSD) across frequencies for conventional and unconventional trials labeled as hypnosis, at the FZ and OZ electrode areas. The data are averaged across participants, with shaded envelopes representing standard deviations. Panels **a** and **c** display z-transformed power values, normalized using the pre-hypnosis baseline (baseline1), highlighting relative changes between conditions. Panels **b** and **d** show the logarithmically transformed power values for both conditions and two pre-hypnosis and post-hypnosis recordings (baseline1 and baseline2), to show absolute power trends across the spectrum.

**Results of the main regression analyses**

Table S1. Regression coefficients for predicting EEG features of interest

|  | b (95% CI) | BF(01) |
| --- | --- | --- |
| FzArea theta power | |  |
| intercept | -0.24 (-1.09, 0.6) | NA |
| induction type - conventional | 0.15 (-0.07, 0.38) | 3.669 |
| label - hypnosis | 0.14 (-0.17, 0.44) | 5.916 |
| expectancy | -0.02 (-0.08, 0.04) | 7.04 |
| gender - male | 0.52 (0.06, 0.97) | 0.583 |
| trial number | 0 (-0.1, 0.1) | 10.869 |
| induction type x label | 0.05 (-0.17, 0.28) | 10.155 |
| OzArea theta power | |  |
| intercept | -0.09 (-0.95, 0.76) | NA |
| induction type - conventional | 0.03 (-0.18, 0.25) | 11.907 |
| label - hypnosis | 0.14 (-0.14, 0.42) | 5.954 |
| expectancy | -0.04 (-0.09, 0.02) | 2.638 |
| gender - male | 0.34 (-0.16, 0.83) | 1.714 |
| trial number | 0.01 (-0.09, 0.11) | 9.325 |
| induction type x label | 0.11 (-0.1, 0.32) | 3.991 |
| FzArea alpha power | |  |
| intercept | 0.22 (-0.62, 1.05) | NA |
| induction type - conventional | -0.13 (-0.31, 0.05) | 4.912 |
| label - hypnosis | -0.28 (-0.53, -0.04) | 0.602 |
| expectancy | 0.04 (-0.01, 0.09) | 1.687 |
| gender - male | 0.07 (-0.44, 0.59) | 3.771 |
| trial number | 0 (-0.08, 0.08) | 12.152 |
| induction type x label | 0.07 (-0.11, 0.24) | 5.95 |
| OzArea alpha power | |  |
| intercept | 0.27 (-0.57, 1.12) | NA |
| induction type - conventional | -0.08 (-0.28, 0.12) | 8.823 |
| label - hypnosis | -0.29 (-0.56, -0.02) | 0.848 |
| expectancy | 0.03 (-0.02, 0.09) | 3.755 |
| gender - male | -0.25 (-0.76, 0.24) | 2.842 |
| trial number | 0 (-0.09, 0.09) | 10.681 |
| induction type x label | 0.03 (-0.17, 0.23) | 8.256 |
| FzArea gamma power | |  |
| intercept | 0.11 (-0.74, 0.98) | NA |
| induction type - conventional | -0.07 (-0.32, 0.17) | 10.597 |
| label - hypnosis | 0.01 (-0.31, 0.33) | 9.342 |
| expectancy | -0.03 (-0.09, 0.04) | 4.946 |
| gender - male | -0.13 (-0.59, 0.32) | 6.442 |
| trial number | 0.01 (-0.1, 0.12) | 9.502 |
| induction type x label | 0.09 (-0.15, 0.32) | 6.306 |
| OzArea gamma power | |  |
| intercept | 0.15 (-0.71, 1.02) | NA |
| induction type - conventional | 0.07 (-0.16, 0.3) | 10.684 |
| label - hypnosis | -0.07 (-0.38, 0.24) | 8.262 |
| expectancy | -0.02 (-0.08, 0.04) | 6.638 |
| gender - male | -0.12 (-0.6, 0.36) | 5.649 |
| trial number | 0.02 (-0.09, 0.12) | 10.735 |
| induction type x label | 0.18 (-0.05, 0.41) | 2.626 |
| connectivity - Oz-PO4 - beta1 band | |  |
| intercept | 0.03 (-0.83, 0.89) | NA |
| induction type - conventional | 0.02 (-0.17, 0.21) | 12.184 |
| label - hypnosis | 0.01 (-0.25, 0.28) | 9.151 |
| expectancy | 0.02 (-0.03, 0.07) | 4.572 |
| gender - male | -0.38 (-0.91, 0.15) | 1.455 |
| trial number | -0.03 (-0.12, 0.06) | 8.044 |
| induction type x label | -0.09 (-0.28, 0.1) | 4.883 |
| connectivity - PzArea-FzArea - alpha2 band | | |
| intercept | 0.16 (-0.68, 1.02) | NA |
| induction type - conventional | -0.11 (-0.29, 0.07) | 6.193 |
| label - hypnosis | -0.03 (-0.28, 0.22) | 8.698 |
| expectancy | 0.01 (-0.05, 0.06) | 6.099 |
| gender - male | -0.11 (-0.65, 0.41) | 3.821 |
| trial number | 0 (-0.08, 0.08) | 9.86 |
| induction type x label | 0.13 (-0.05, 0.31) | 2.876 |
| connectivity - O1-PZ - theta band | |  |
| intercept | 0.1 (-0.74, 0.96) | NA |
| induction type - conventional | -0.23 (-0.42, -0.05) | 0.586 |
| label - hypnosis | 0.15 (-0.09, 0.4) | 4.364 |
| expectancy | -0.04 (-0.1, 0.01) | 1.65 |
| gender - male | -0.1 (-0.65, 0.43) | 4.349 |
| trial number | 0 (-0.08, 0.08) | 10.341 |
| induction type x label | 0.09 (-0.09, 0.28) | 5.273 |

Note: b indicates posterior mode of the regression coefficient based on 1 chain and 10000 iterations, 95% CI represents 95% credible intervals. The table is segmented based on the dependent variable used in the model.

Table S2. Regression coefficients for predicting EEG features of interest - with hypnotizability added as a predictor

|  | b (95% CI) | BF(01) | |
| --- | --- | --- | --- |
| hypnosis depth |  |  | |
| intercept | 2.8 (0.31, 5.27) | NA | |
| induction type - conventional | 0.86 (0.16, 1.57) | 0.7 | |
| label - hypnosis | 1.33 (0.37, 2.28) | 0.24 | |
| expectancy | 0.28 (0.09, 0.46) | 0.06 | |
| gender - male | 0.33 (-0.93, 1.62) | 3.77 | |
| trial number | -0.11 (-0.44, 0.21) | 4.56 | |
| hypnotizability | 0.38 (0.13, 0.62) | 0.04 | |
| induction type x label | -0.04 (-0.76, 0.66) | 5.37 | |
| FzArea theta power | |  | |
| intercept | -0.32 (-1.22, 0.58) | NA | |
| induction type - conventional | 0.12 (-0.12, 0.35) | 7.06 | |
| label - hypnosis | 0.13 (-0.18, 0.44) | 6.22 | |
| expectancy | -0.02 (-0.09, 0.04) | 4.01 | |
| gender - male | 0.5 (-0.02, 1) | 0.77 | |
| trial number | 0.05 (-0.06, 0.15) | 5.23 | |
| hypnotizability | 0 (-0.09, 0.09) | 5.07 | |
| induction type x label | -0.06 (-0.29, 0.17) | 6.88 | |
| OzArea theta power | |  | |
| intercept | -0.26 (-1.09, 0.57) | NA | |
| induction type - conventional | 0.06 (-0.17, 0.29) | 8.57 | |
| label - hypnosis | 0.28 (-0.02, 0.58) | 1.63 | |
| expectancy | -0.06 (-0.12, 0) | 0.77 | |
| gender - male | 0.34 (-0.1, 0.79) | 1.93 | |
| trial number | 0.02 (-0.08, 0.12) | 8.47 | |
| hypnotizability | 0.07 (-0.01, 0.16) | 1.05 | |
| induction type x label | 0.12 (-0.11, 0.35) | 5.01 | |
| FzArea alpha power | |  | |
| intercept | 0.12 (-0.77, 1.02) | NA | |
| induction type - conventional | -0.1 (-0.28, 0.07) | 5.45 | |
| label - hypnosis | -0.2 (-0.45, 0.05) | 2.07 | |
| expectancy | 0.03 (-0.02, 0.08) | 3.38 | |
| gender - male | -0.07 (-0.65, 0.5) | 3.53 | |
| trial number | 0.04 (-0.05, 0.12) | 5.37 | |
| hypnotizability | -0.04 (-0.15, 0.07) | 2.04 | |
| induction type x label | 0.03 (-0.15, 0.21) | 7.99 | |
| OzArea alpha power | |  | |
| intercept | 0.12 (-0.78, 1.06) | NA | |
| induction type - conventional | -0.02 (-0.23, 0.19) | 6.13 | |
| label - hypnosis | -0.2 (-0.48, 0.09) | 1.86 | |
| expectancy | 0.04 (-0.02, 0.09) | 2.48 | |
| gender - male | -0.34 (-0.91, 0.19) | 2.24 | |
| trial number | 0.02 (-0.07, 0.12) | 8.12 | |
| hypnotizability | -0.02 (-0.12, 0.09) | 3.5 | |
| induction type x label | 0.01 (-0.2, 0.23) | 15.3 | |
| FzArea gamma power | |  | |
| intercept | -0.02 (-0.9, 0.84) | NA | |
| induction type - conventional | -0.01 (-0.24, 0.23) | 10.25 | |
| label - hypnosis | 0.01 (-0.3, 0.33) | 8.23 | |
| expectancy | -0.01 (-0.08, 0.05) | 4.49 | |
| gender - male | -0.12 (-0.59, 0.35) | 4.58 | |
| trial number | -0.02 (-0.13, 0.09) | 7.3 | |
| hypnotizability | -0.02 (-0.11, 0.07) | 3.76 | |
| induction type x label | 0.12 (-0.12, 0.36) | 4.85 | |
| OzArea gamma power | |  | |
| intercept | -0.04 (-0.86, 0.78) | NA | |
| induction type - conventional | 0.05 (-0.19, 0.28) | 9.77 | |
| label - hypnosis | -0.02 (-0.34, 0.29) | 8.18 | |
| expectancy | -0.02 (-0.08, 0.04) | 4.44 | |
| gender - male | 0 (-0.43, 0.4) | 6.8 | |
| trial number | 0.02 (-0.09, 0.13) | 7.59 | |
| hypnotizability | -0.02 (-0.1, 0.05) | 4.35 | |
| induction type x label | 0.08 (-0.15, 0.32) | 6.38 | |
| connectivity - Oz-PO4 - beta1 band | |  | |
| intercept | 0.13 (-0.75, 1.04) | NA | |
| induction type - conventional | 0.04 (-0.16, 0.25) | 10.93 | |
| label - hypnosis | 0.02 (-0.27, 0.3) | 8.01 | |
| expectancy | 0.02 (-0.03, 0.08) | 7.34 | |
| gender - male | -0.44 (-0.98, 0.08) | 1.09 | |
| trial number | -0.02 (-0.11, 0.08) | 7.16 | |
| hypnotizability | -0.02 (-0.11, 0.08) | 3.13 | |
| induction type x label | -0.07 (-0.27, 0.14) | 5.9 | |
| connectivity - PzArea-FzArea - alpha2 band | | |  |
| intercept | 0.12 (-0.82, 1.05) | NA | |
| induction type - conventional | -0.12 (-0.32, 0.06) | 5.15 | |
| label - hypnosis | -0.04 (-0.3, 0.23) | 7.81 | |
| expectancy | 0.01 (-0.05, 0.06) | 4.62 | |
| gender - male | -0.14 (-0.74, 0.45) | 3 | |
| trial number | -0.01 (-0.1, 0.08) | 7.39 | |
| hypnotizability | -0.05 (-0.16, 0.06) | 2.05 | |
| induction type x label | 0.09 (-0.1, 0.28) | 4.66 | |
| connectivity - O1-PZ - theta band | |  | |
| intercept | 0.04 (-0.87, 0.96) | NA | |
| induction type - conventional | -0.16 (-0.36, 0.03) | 3.1 | |
| label - hypnosis | 0.21 (-0.07, 0.49) | 2.63 | |
| expectancy | -0.05 (-0.1, 0.01) | 1.19 | |
| gender - male | -0.1 (-0.66, 0.46) | 3.81 | |
| trial number | 0.02 (-0.07, 0.11) | 8.59 | |
| hypnotizability | 0.02 (-0.08, 0.13) | 3.36 | |
| induction type x label | 0.1 (-0.1, 0.3) | 5.09 | |

Note: b indicates posterior mode of the regression coefficient based on 1 chain and 10000 iterations, 95% CI represents 95% credible intervals. The table is segmented based on the dependent variable used in the model.

**Procedure-level EEG changes**

Table S3. Procedure-level EEG changes

|  | mode (95% CI) | BF(01) |
| --- | --- | --- |
| procedure type: whitenoise |  |  |
| FzArea theta power | -0.16 (-0.42, 0.14) | 3.96 |
| OzArea theta power | -0.19 (-0.5, 0.13) | 3.53 |
| FzArea alpha power | 0.07 (-0.28, 0.41) | 6.47 |
| OzArea alpha power | -0.03 (-0.32, 0.28) | 6.88 |
| FzArea gamma power | -0.25 (-0.5, 0.04) | 1.49 |
| OzArea gamma power | -0.15 (-0.39, 0.1) | 3.45 |
| connectivity - Oz-PO4 - beta1 band | 0.22 (-0.17, 0.57) | 3.66 |
| connectivity - PzArea-FzArea - alpha2 band | 0.24 (-0.05, 0.52) | 1.79 |
| connectivity - O1-PZ - theta band | 0.05 (-0.33, 0.44) | 6.74 |
| procedure type: relaxation |  |  |
| FzArea theta power | 0.16 (-0.31, 0.61) | 4.49 |
| OzArea theta power | 0.11 (-0.36, 0.57) | 5.03 |
| FzArea alpha power | -0.34 (-0.68, 0.06) | 1.35 |
| OzArea alpha power | -0.32 (-0.69, 0.1) | 1.75 |
| FzArea gamma power | -0.31 (-0.6, 0.03) | 1.05 |
| OzArea gamma power | -0.28 (-0.44, -0.08) | 0.15 |
| connectivity - Oz-PO4 - beta1 band | -0.09 (-0.59, 0.41) | 5.37 |
| connectivity - PzArea-FzArea - alpha2 band | -0.29 (-0.89, 0.37) | 3.84 |
| connectivity - O1-PZ - theta band | 0.11 (-0.43, 0.62) | 5.29 |
| procedure type: confusion |  |  |
| FzArea theta power | 0.11 (-0.32, 0.53) | 5.93 |
| OzArea theta power | 0 (-0.4, 0.41) | 6.62 |
| FzArea alpha power | 0.06 (-0.37, 0.48) | 6.5 |
| OzArea alpha power | 0 (-0.41, 0.41) | 6.5 |
| FzArea gamma power | 0.18 (-0.26, 0.58) | 4.83 |
| OzArea gamma power | 0.32 (-0.25, 0.84) | 3.51 |
| connectivity - Oz-PO4 - beta1 band | 0.06 (-0.33, 0.44) | 6.43 |
| connectivity - PzArea-FzArea - alpha2 band | 0.22 (-0.01, 0.42) | 1.01 |
| connectivity - O1-PZ - theta band | -0.21 (-0.56, 0.17) | 3.75 |
| procedure type: embedded |  |  |
| FzArea theta power | 0.14 (-0.56, 0.8) | 4.92 |
| OzArea theta power | 0.2 (-0.49, 0.84) | 4.41 |
| FzArea alpha power | -0.25 (-0.67, 0.21) | 3.13 |
| OzArea alpha power | -0.14 (-0.54, 0.3) | 4.46 |
| FzArea gamma power | 0.3 (-0.54, 1.07) | 4.26 |
| OzArea gamma power | -0.27 (-0.41, -0.08) | 0.09 |
| connectivity - Oz-PO4 - beta1 band | -0.09 (-0.56, 0.41) | 5.12 |
| connectivity - PzArea-FzArea - alpha2 band | -0.44 (-0.99, 0.18) | 1.94 |
| connectivity - O1-PZ - theta band | 0.2 (-0.31, 0.67) | 3.73 |

Note: mode indicates posterior mode of the change in EEG features of interest based on 1 chain and 10000 iterations, 95% CI represents 95% credible intervals. The table is segmented based on the induction procedure type, and only contains data from trials labeled as hypnosis.

**Prediction accuracy of EEG features in combined**

We also built a mixed effect logistic regression model to see if the EEG features of interest are informative in predicting which induction type (conventional or unconventional) was used in the trial.

The model included all EEG features of interest as fixed effect predictors, and participant ID as random intercept. We only looked at trials where the procedure was labeled as hypnosis, and only 70 out of the 92 trials labeled as hypnosis were used in this analysis due to outliers. The model was able to correctly classify 64% of the trials (base rate of conventional induction was 51%). 68% of the conventional inductions, and 58% of the unconventional induction trials were classified correctly. While this prediction accuracy is slightly better than the base rate, due to the large number of predictors, considerable overfitting is possible. The permutation analysis confirms this, since the average accuracy of correctly classifying conventional induction trials was 70%, and the average accuracy of correctly classifying unconventional induction trials was 64%, both slightly higher than that of the original model. This indicates that taken together, the EEG features of interest are not helpful at distinguishing between conventional and unconventional inductions. That is, the electrophysiological signature of conventional and unconventional inductions were comparable, at least on the EEG features selected in this study based on previous literature. Bayesian evidence was inconclusive about the usefulness of these predictors (BF01 = 0.94). Since the Bayes Factor was very close to 1, the model including the EEG features as predictors and the null model with no EEG features is roughly equally as likely to be true, more data is needed to distinguish between the likelihood of the two models.

**Correlation of EEG changes with hypnosis depth and hypnotizability.**

Table S4. Correlation of hypnosis depth and hypnotizability with change in EEG power

| Power band | Induction type | Hypnosis depth | | Hypnotizability | |
| --- | --- | --- | --- | --- | --- |
|  |  | Frontal | Occipital | Frontal | Occipital |
| theta | conventional | 0.03 [-0.26, 0.31]a | 0.02 [-0.27, 0.31]a | 0.01 [-0.3, 0.32]a | 0.28 [-0.03, 0.54] |
|  | unconventional | 0.04 [-0.25, 0.32]a | 0.15 [-0.15, 0.42]a | 0.05 [-0.26, 0.36]a | 0.28 [-0.04, 0.55] |
| alpha | conventional | -0.01 [-0.29, 0.27]a | 0.02 [-0.27, 0.3]a | -0.19 [-0.47, 0.13] | -0.2 [-0.48, 0.12] |
|  | unconventional | -0.08 [-0.35, 0.21]a | -0.08 [-0.36, 0.2]a | -0.14 [-0.43, 0.18]a | -0.07 [-0.38, 0.25]a |
| gamma | conventional | -0.17 [-0.44, 0.11] | 0.23 [-0.06, 0.49] | -0.23 [-0.5, 0.09] | -0.01 [-0.32, 0.3]a |
|  | unconventional | -0.27 [-0.51, 0] | 0.14 [-0.17, 0.41]a | 0.07 [-0.24, 0.38]a | 0.15 [-0.18, 0.45]a |

Note: Values represent rho, the estimate of the true linear correlation coefficient and 95% credible intervals. a: 3 < BF01 < 10 indicating moderate Bayesian evidence supporting no correlation.

Table S5. Correlation of hypnosis depth and hypnotizability with change in functional connectivity

| Power band: connected regions | Induction type | Hypnosis depth | Hypnotizability |
| --- | --- | --- | --- |
| beta1: OZ-PO4 | conventional | -0.15 [-0.41, 0.15]a | -0.15 [-0.43, 0.16]a |
|  | unconventional | -0.12 [-0.4, 0.17]a | 0.00 [-0.31, 0.31]a |
| alpha2: PZ-FZ | conventional | -0.09 [-0.37, 0.19]a | -0.03 [-0.33, 0.28]a |
|  | unconventional | -0.13 [-0.4, 0.17]a | -0.19 [-0.47, 0.13] |
| theta: O1-PZ | conventional | 0 [-0.28, 0.29]a | 0.06 [-0.25, 0.36]a |
|  | unconventional | 0.04 [-0.26, 0.33]a | 0.07 [-0.25, 0.37]a |

Note: Values represent rho, the estimate of the true linear correlation coefficient and 95% credible intervals. a: 3 < BF01 < 10 indicating moderate Bayesian evidence supporting no correlation.

We also built linear regression models to predict hypnosis depth and hypnotizability by including all EEG features of interest as predictors in trials where the procedure was labeled as hypnosis. We fit separate models on data from conventional and unconventional induction trials. The model predicting hypnosis depth using the EEG features of interest built on the data from conventional induction trials had a better fit (R² = 0.32) than the one built on the data from the unconventional induction trials (R² = 0.19). Although the difference in R² values might seem substantial, neither of these models were significantly better than the permutation models. The averaged R² of the permutation models on the conventional hypnosis trial dataset was 0.23, with 17% of the permutation models producing better R² than the original model. The averaged R² of the permutation models on the unconventional hypnosis trial dataset was also 0.23, with 63% of the permutation models producing better R² than the original model. Very strong Bayesian evidence supported the null models, indicating that the model containing all EEG features of interest is not informative in predicting hypnosis depth (conventional induction condition: BF01 = 276.32, unconventional induction condition: BF01 = 4477.21).

The models performed even worse when trying to predict hypnotizability. In these cases the model built on the unconventional induction datasets performed slightly better (R² = 0.19) than that built on the conventional induction data (R² = 0.12). However, the average permutation model performance was better in both cases (R² = 0.28 in both cases). Again, very strong Bayesian evidence supported the null models (conventional induction condition: BF01 = 15359.81, unconventional induction condition: BF01 = 4189.75).

These results indicate that the EEG features did not seem to have a substantial predictive power in predicting hypnosis depth or hypnotizability, regardless of the type of induction used.

**Deception effectiveness and sensitivity analysis**

29 (63%) of the 46 participants indicated that they suspect that one of the two techniques labeled as hypnosis was not hypnosis. However, when prompted to guess which of the two inductions was real, only 18 (62%) out of these 29 were correct (despite having 0.5 probability to get this right just by chance). 30 (65%) participants reported that they suspected one of the two techniques labeled as control was hypnosis, with 24 (80%) of these 30 being correct at identifying which of the two was the real hypnotic induction. Thus, we determined that 28 (61%) participants were deceived by the placebo manipulation, and 22 (48%) deceived by the fake control trials. Embedded hypnosis was selected as being the real hypnosis 14 (30%) out of the 46 participants, while white noise hypnosis was selected as being the real hypnosis 18 (38%) of the participants, indicating that white noise hypnosis might be a slightly more convincing induction technique.

In order to assess the effect of unsuccessful deception on our conclusions, we conducted a sensitivity analysis by rerunning our main analyses on the subsample of trials in which participants appeared to be deceived by the deception. This consists of 100 (54%) trials. Results of the analysis about regression coefficients for predicting hypnotic depth and EEG features of interest in this subsample of trials are displayed in Table S5.

Table S6. Regression coefficients for predicting hypnotic depth and EEG features of interest

|  | b (95% CI) | BF(01) |
| --- | --- | --- |
| hypnosis depth |  |  |
| intercept | 2.92 (0.03, 5.78) | NA |
| induction type - conventional | 0.28 (-0.5, 1.06) | 6.4 |
| label - hypnosis | 2.18 (1.01, 3.35) | 0.01 |
| expectancy | 0.36 (0.14, 0.58) | 0.03 |
| gender - male | -1.06 (-2.55, 0.46) | 1.75 |
| trial number | -0.06 (-0.44, 0.31) | 7.66 |
| induction type x label | 0.16 (-0.63, 0.91) | 6.08 |
| FzArea theta power | |  |
| intercept | -0.18 (-1.34, 0.99) | NA |
| induction type - conventional | 0.17 (-0.14, 0.49) | 3.9 |
| label - hypnosis | 0.06 (-0.41, 0.52) | 5.62 |
| expectancy | -0.01 (-0.1, 0.07) | 5.28 |
| gender - male | 0.39 (-0.21, 1) | 2.01 |
| trial number | 0.04 (-0.11, 0.2) | 6.86 |
| induction type x label | 0.18 (-0.13, 0.48) | 3.65 |
| OzArea theta power | |  |
| intercept | 0.01 (-1.14, 1.16) | NA |
| induction type - conventional | 0.01 (-0.29, 0.33) | 8.47 |
| label - hypnosis | 0.18 (-0.29, 0.64) | 4.62 |
| expectancy | -0.04 (-0.12, 0.04) | 3.2 |
| gender - male | 0.26 (-0.33, 0.85) | 3.31 |
| trial number | 0.08 (-0.07, 0.23) | 4.72 |
| induction type x label | 0.34 (0.03, 0.66) | 0.77 |
| FzArea alpha power | |  |
| intercept | -0.07 (-1.04, 0.92) | NA |
| induction type - conventional | 0.02 (-0.19, 0.23) | 8.29 |
| label - hypnosis | -0.1 (-0.43, 0.22) | 4.37 |
| expectancy | 0.01 (-0.05, 0.07) | 5.61 |
| gender - male | -0.19 (-0.79, 0.39) | 2.89 |
| trial number | -0.04 (-0.14, 0.07) | 6.47 |
| induction type x label | 0.07 (-0.14, 0.27) | 5.65 |
| OzArea alpha power | |  |
| intercept | 0.06 (-0.99, 1.14) | NA |
| induction type - conventional | -0.02 (-0.24, 0.2) | 8.3 |
| label - hypnosis | -0.24 (-0.59, 0.09) | 2 |
| expectancy | 0.01 (-0.06, 0.07) | 4.09 |
| gender - male | -0.29 (-0.95, 0.35) | 1.86 |
| trial number | -0.02 (-0.12, 0.09) | 7.3 |
| induction type x label | -0.01 (-0.23, 0.21) | 5.77 |
| FzArea gamma power | |  |
| intercept | -0.05 (-1.26, 1.13) | NA |
| induction type - conventional | -0.18 (-0.51, 0.14) | 4.76 |
| label - hypnosis | 0.17 (-0.3, 0.65) | 5.43 |
| expectancy | -0.08 (-0.16, 0.01) | 1.15 |
| gender - male | -0.13 (-0.74, 0.49) | 5.4 |
| trial number | 0.03 (-0.13, 0.18) | 7.67 |
| induction type x label | 0 (-0.32, 0.32) | 6.5 |
| OzArea gamma power | |  |
| intercept | 0.24 (-0.91, 1.4) | NA |
| induction type - conventional | -0.04 (-0.3, 0.23) | 8.74 |
| label - hypnosis | -0.2 (-0.61, 0.2) | 3.64 |
| expectancy | 0 (-0.08, 0.07) | 5.16 |
| gender - male | -0.23 (-0.92, 0.42) | 2.97 |
| trial number | 0.06 (-0.07, 0.19) | 5.05 |
| induction type x label | 0.09 (-0.16, 0.34) | 5.05 |
| connectivity - Oz-PO4 - beta1 band | |  |
| intercept | 0.22 (-0.89, 1.33) | NA |
| induction type - conventional | -0.01 (-0.28, 0.25) | 8.6 |
| label - hypnosis | -0.13 (-0.55, 0.3) | 4.82 |
| expectancy | 0.02 (-0.05, 0.1) | 4.52 |
| gender - male | -0.4 (-1.02, 0.21) | 1.72 |
| trial number | -0.03 (-0.15, 0.11) | 7.46 |
| induction type x label | -0.02 (-0.29, 0.24) | 6.55 |
| connectivity - PzArea-FzArea - alpha2 band | | |
| intercept | 0 (-1.13, 1.12) | NA |
| induction type - conventional | -0.13 (-0.38, 0.11) | 5.9 |
| label - hypnosis | -0.05 (-0.44, 0.35) | 6.05 |
| expectancy | 0.02 (-0.06, 0.1) | 3.8 |
| gender - male | -0.11 (-0.77, 0.54) | 3.02 |
| trial number | -0.09 (-0.21, 0.03) | 2.56 |
| induction type x label | 0.1 (-0.15, 0.35) | 4.06 |
| connectivity - O1-PZ - theta band | |  |
| intercept | 0.17 (-0.9, 1.26) | NA |
| induction type - conventional | -0.3 (-0.52, -0.09) | 0.37 |
| label - hypnosis | 0.14 (-0.21, 0.48) | 4.07 |
| expectancy | -0.05 (-0.11, 0.02) | 1.95 |
| gender - male | -0.13 (-0.82, 0.5) | 3.25 |
| trial number | 0.05 (-0.06, 0.15) | 6.03 |
| induction type x label | 0.16 (-0.06, 0.38) | 2.45 |

Note: b indicates posterior mode of the regression coefficient based on 1 chain and 10000 iterations, 95% CI represents 95% credible intervals. The table is segmented based on the dependent variable used in the model.
